# A single-arm open-label pilot study of brief mindfulness meditation to control impulsivity in Parkinson’s disease

**DOI:** 10.1371/journal.pone.0266354

**Published:** 2022-04-06

**Authors:** Jinsoo Koh, Maiko Takahashi, Yasuhiko Ohmae, Junko Taruya, Mayumi Sakata, Masaaki Yasui, Masaki Terada, Hidefumi Ito

**Affiliations:** 1 Department of Neurology, Wakayama Medical University, Wakayama, Wakayama Prefecture, Japan; 2 Wakayama-Minami Radiology Clinic, Wakayama, Wakayama Prefecture, Japan; Utano National Hospital, JAPAN

## Abstract

**Background:**

Impulse control disorders are detrimental neuropsychiatric symptoms of Parkinson’s disease. Increased impulsivity is a predisposing factor for impulse control disorders and should therefore be controlled. Recently, mindfulness meditation as a non-drug therapy has been reported to be useful in improving neuropsychiatric symptoms, such as impulsivity.

**Methods:**

We performed a prospective single-arm, open-label pilot trial to investigate the effectiveness of mindfulness meditation to control impulsivity in patients with Parkinson’s disease (UMIN clinical trials registry: UMIN000037779).

**Results:**

Twenty patients with Parkinson’s disease were enrolled in an 8-week mindfulness meditation program. As a primary outcome, we investigated whether the score of the Barratt Impulsiveness Scale (BIS-11) was significantly reduced after the intervention. As an exploratory examination, functional connectivity changes were also assessed by resting-state functional magnetic resonance imaging. After the intervention, the BIS-11 score was decreased from 59.5 [55.6, 63.3] (mean [95% confidence interval]) to 55.2 [50.3, 60.1] (ΔBIS-11: -4.2, [-7.5, -0.9]). Functional connectivity was increased in the default mode network (DMN) at a cluster including the precuneus, posterior cingulate gyrus, and left posterior lobe (false discovery rate-adjusted p [FDR-p] = 0.046) and in the right frontoparietal network (FPN) at the medial frontal lobe (FDR-p = 0.039).

**Conclusions:**

This open-label, single-arm pilot study provided preliminary data for mindfulness meditation to control the impulsivity of patients with PD. A brief mindfulness meditation program may be effective in controlling impulsivity in PD and may change the functional connectivity of the DMN and right FPN.

## Introduction

Parkinson’s disease (PD) is the most common neurodegenerative motor disease. It is estimated that the number of affected patients will increase from 6.9 million in 2015 to 14.2 million in 2040 [1]. Recently, it has been emphasized that it is important to deal not only with the motor symptoms of PD but also with the non-motor symptoms, such as neuropsychiatric, cognitive, and autonomic symptoms and sleep disorders. Among them, impulse control disorders (ICDs) are common neuropsychiatric symptoms that can lead to unproductive, harmful, and even illegal activity [2]. ICDs and related disorders include pathological gambling, hypersexuality, binge eating, compulsive shopping, punding, hobbyism, and dopamine dysregulation syndrome. Although the pathophysiology of ICDs has not been clarified, hyperstimulation of the mesolimbic dopaminergic pathway associated with dopamine replacement therapy [3,4], hyperglycemic metabolism in the frontal lobe [5], and altered functional connectivity in the cortico-striato-limbic circuit [6,7] have been suggested to have important roles. Recently, trait impulsivity without an apparent ICD in PD patients has been reported in several studies [8–10]. High impulsivity is a precursor of ICDs and can also lead to the development of severe ICDs [11,12]. Therefore, it is assumed that controlling impulsivity might prevent or reduce the severity of ICDs.

Some clinical trials aiming to treat ICDs have been conducted. A small group trial of amantadine was reported as effective [13], but an epidemiological study showed the opposite result [2]. Naltrexone, selective serotonin reuptake inhibitors, and clozapine were not shown to be effective [14,15]. Reducing dopamine replacement therapy was reported to improve some ICDs, such as pathological gambling, hypersexuality, binge eating, and compulsive shopping, but did not improve punding [16] and can cause deterioration of motor symptoms or dopamine agonist withdrawal syndrome. Conversely, a cognitive-behavioral therapy-based intervention was recently shown to have a positive effect as a non-drug treatment for ICDs [17].

Among non-drug therapies, mindfulness meditation is a simple and feasible approach. Mindfulness is defined as “maintaining attention on the immediate experience and maintaining an attitude of acceptance toward this experience” [18]. An increasing number of reports have investigated the effectiveness of mindfulness meditation for various psycho-cognitive conditions [19]. In particular, evidence for an effect of mindfulness meditation on depression has been established [20,21]. Recently, a randomized trial of mindfulness yoga showed that it improved depression in patients with PD [22]. Besides depression, mindfulness meditation can improve attention deficit hyperactivity disorder [23] and drug addiction [24] and can reduce impulsivity in middle- and high-school students [25]. Although longer practices of mindfulness meditation are more effective, brief versions have also been shown to be effective [26,27]. Brief mindfulness meditation has the advantage of being easy to get started and continue, especially in patients with neurodegenerative diseases. Herein, we performed a single-arm pilot trial to investigate whether mindfulness meditation could be used to control the impulsivity of patients with PD.

In addition, we examined the structural and functional changes before and after mindfulness meditation in PD patients. A previous study indicated that mindfulness meditation resulted in structural changes in the amygdala and hippocampus of PD patients [28]. However, to the best of our knowledge, no reports have examined the changes in functional connectivity caused by mindfulness meditation in PD patients. Conversely, altered functional connectivity has been detected in relation to impulsivity or ICDs in PD [6,7]. Trait mindfulness of healthy volunteers, which was assessed with the Mindful Attention Awareness Scale (MAAS), was also shown to be correlated with functional connectivity among the default mode network (DMN), salience network (SN), and frontoparietal network (FPN) [29]. Furthermore, mindfulness meditation can reportedly be used to control a hyperactivated DMN [18]. Therefore, mindfulness meditation might control the impulsivity of PD patients by modulating functional connectivity. In this study, we showed that a brief course of mindfulness meditation could change the functional connectivity of patients with PD and discussed how it was associated with impulsivity.

## Material and methods

### Study design, registration, and consent

This study was an 8-week, single-arm, open-label clinical trial of mindfulness meditation to control impulsivity in patients with PD. The setting was outpatient clinics. Assessments were conducted at baseline (T0), week 2 (T1), and week 8 (T2) (S1 Fig). This study was conducted according to the principles of the Declaration of Helsinki and approved by the Wakayama Medical University Ethics Committee (approval number: 2698). All participants provided written informed consent. This study is registered at the UMIN clinical trials registry (UMIN000037779).

### Participants

Twenty patients with idiopathic PD were enrolled using convenience sampling from September 1, 2019, to April 31, 2020. The participants were recruited from outpatients of our institute. We included clinically established or probable PD diagnosed by the clinical diagnostic criteria of the Movement Disorder Society. All participants were older than 19 years and could give written consent. We excluded participants who could not conduct mindfulness meditation because of severe dementia (Mini-Mental State Examination score < 20), hearing loss, visual disorder, or confusion; who were currently participating in another clinical trial or meditation/yoga program; who had changed anti-parkinsonian drugs within 28 days; who received new pharmacologic or cognitive-behavioral therapy within 84 days; or with current stroke or encephalitis. Cases with involuntary movements affecting functional magnetic resonance imaging (fMRI) were also excluded.

### Mindfulness meditation

The participants received a weekly 45-min group session of mindfulness meditation for 8 weeks by a professional clinical psychologist (Y.O.), and were required to practice meditation daily for 15 min on 6 days or more per week. Mindfulness meditation included body scan, mindfulness movements, and controlled breathing [27,30]. The participants were given instruction papers in each group session for daily home-based practice, and filled in an implementation record. In addition to regular meditation, the participants also received instructions on meditation techniques during daily activities such as eating meditation and walking meditation. During the period from registration to the final evaluation, the participants were prohibited from changing antiparkinsonian, antipsychotic, or antidepressant drugs.

### Outcomes and assessments

Outcome measures were conducted by a face-to-face clinical assessment and interview. To investigate the hypothesis that meditation improves the impulsivity of PD, we set the difference in the Barratt Impulsiveness Scale 11th version (ΔBIS-11) score [31] between T0 and T2 as the primary outcome. BIS-11 is the most commonly used self-reported questionnaire that measures the subdomains of attentional, motor, and non-planning impulsivity [32]. Secondary outcomes consisted of the Questionnaire for Impulsive Compulsive Disorders in PD-Rating Scale (QUIP-RS) [33], Hospital Anxiety and Depression Scale (HADS) [34], Parkinson’s Disease Questionnaire-8 (PDQ-8) [35], MAAS [36], Neuropsychiatric Inventory-Questionnaire (NPI-Q) [37], and Movement Disorder Society Unified Parkinson’s Disease Rating Scale (MDS-UPDRS) motor score (part 3) [38]. MDS-UPDRS was rated at T0 and T2, and the other scales were rated at T0, T1, and T2. All assessments were conducted in the “ON” state with usual medication.

### Statistical analysis of clinical, motor, and neuropsychological data

All patients allocated to the mindfulness meditation program were analyzed. Patient characteristics were analyzed using JMP Pro14 software. Normality of the data was assessed using the Shapiro-Wilk test. Categorical variables are presented as numerals. Normally distributed continuous variables are presented as means and standard deviation. Non-normally distributed continuous variables are presented as medians and interquartile range. The difference of each outcome between T2 and T0 is shown by the mean [95% confidence interval (CI)] and p-value by a one-sample *t*-test as a reference value. The Wilcoxon signed-rank test was performed for non-normally distributed variables. Pearson’s correlation coefficient was used to investigate the relationship between each parameter.

### MRI acquisition

MRI was performed on each participant at T0 and T2 using a 3-Tesla MRI scanner with a 32-channel head coil. To minimize motion artefacts, the patients underwent MRI during the “ON” state while taking their usual medications, as in previous studies [6,39]. The following parameters were used to acquire T1-weighted structural images: TR = 7 ms, TE = 3.3 ms, field of view (FOV) = 220 mm, matrix scan = 256, slice thickness = 0.9 mm, and flip angle = 10°. The following parameters were used for functional data using a gradient-echo echo-planar pulse sequence sensitive to blood oxygen level-dependent contrast: TR = 3000 ms, TE = 30 ms, FOV = 192 mm, matrix scan = 64, slice thickness = 3 mm, number of slices 38, and flip angle = 80°, which covered the entire cerebral gray matter and cerebellum. Three runs, each with 105 volumes, were performed on each participant. In total, data were acquired from each participant over approximately 15 min in the resting state, as this duration was deemed the most appropriate to obtain reliable data [40,41]. The participants were asked to remain awake, but keep their eyes closed during acquisition.

#### fMRI analysis

We used Statistical Parametric Mapping software, version 12 (SPM12) and CONN toolbox, version 17 in MATLAB R2014a for MRI data preprocessing. The CONN toolbox is designed to preprocess functional and anatomical files, including realignment, slice-timing correction, outlier identification, co-registration, segmentation, normalization, and smoothing [42]. Preprocessing of functional data was performed by the default preprocessing pipeline. The initial five scans were removed to eliminate equilibration effects. The images were realigned and unwarped. Slice-timing was corrected in the ascending order. For functional outlier detection, we used intermediate settings (97th percentile in normative samples) with adaptive resonance theory implemented in CONN. With a series of three runs of functional imaging, we analyzed a total of 300 functional images per subject per session. Functional and structural data were segmented to gray matter, white matter, and cerebrospinal fluid, and normalized to Montreal Neurological Institute coordinates. Functional images were smoothed to an 8 mm full width at half maximum Gaussian kernel. Subsequently, denoising was performed by default, including band-pass filtering (0.008–0.09 Hz) and linear regression.

For 1st-level analysis, we used group-independent component analysis (ICA) with default settings: the number of factors was 40, G1 Fast ICA + GICA3 back-projection, and dimensionality reduction was 64. The DMN, SN, and FPN were identified using implemented ICA tools with visual inspection. For 2nd-level analysis, a paired *t*-test was performed on the differences in spatial properties between T0 and T2 for the DMN, SN, and FPN. A cluster-size false discovery rate adjusted p (FDR-p) < 0.05 was considered significant.

#### Structural MRI analysis

For voxel-based morphometry, we used SPM12 in MATLAB. T1-weighted structural images were segmented to gray matter, white matter, cerebrospinal fluid, and other non-brain portions. Spatial normalization was performed on gray matter probability maps using the diffeomorphic anatomical registration with an exponentiated Lie algebra algorithm. Normalized imaging was smoothed using an 8 mm full width at half maximum Gaussian kernel. Age and sex were included as nuisance covariates. We applied total brain volume for global calculations. Implicit and explicit masking were used. To evaluate differences between T0 and T2, we performed a paired *t*-test, and family-wise error corrected p < 0.05 was considered significant.

## Results

### Patients

The demographic data of the 20 PD patients are shown in Table 1. There were no protocol deviations. All PD patients had a Mini-Mental State Examination score ≥ 23. A QUIP-RS score of 6 or higher, which is a cutoff score of the Japanese version of QUIP-RS [43], was observed in 14 participants at baseline and in 6 participants after the mindfulness program. At baseline, the MAAS score was positively correlated with the Mini-Mental State Examination score (r = 0.54, p = 0.013), and negatively correlated with the BIS-11 score (r = -0.56, p = 0.011). Although not statistically significant, the QUIP-RS score also tended to be negatively correlated with the MAAS score (r = -0.43, p = 0.06). The other parameters had no significant correlations with the MAAS score.

**Table 1:** Demographic and clinical parameters of patients with Parkinson’s disease.

	N = 20
**Age, years**	69.0 ± 5.0
**Sex, female/male**	9/11
**Disease duration, years**	5 (3–9.5)
**Levodopa, mg**	300 (225–437.5)
**Dopamine agonist, mg/LEDD**	62.5 (0–171)
**MMSE**	30 (27.3–30)
**HY, 1/2/3**	8/11/1
**BIS−11**	59.5 ± 8.2
** Attentional**	14.7 ± 3.8
** Motor**	19.9 ± 3.4
** Non-planning**	25.0 ± 4.7

Data are presented as the mean ± standard deviation or median (interquartile range).

BIS-11, Barratt Impulsiveness Scale 11th version; HY, Hoehn and Yahr stage; LEDD, Levodopa Equivalent Daily Dose; MMSE, Mini-Mental State Examination.

**Fig 1:**
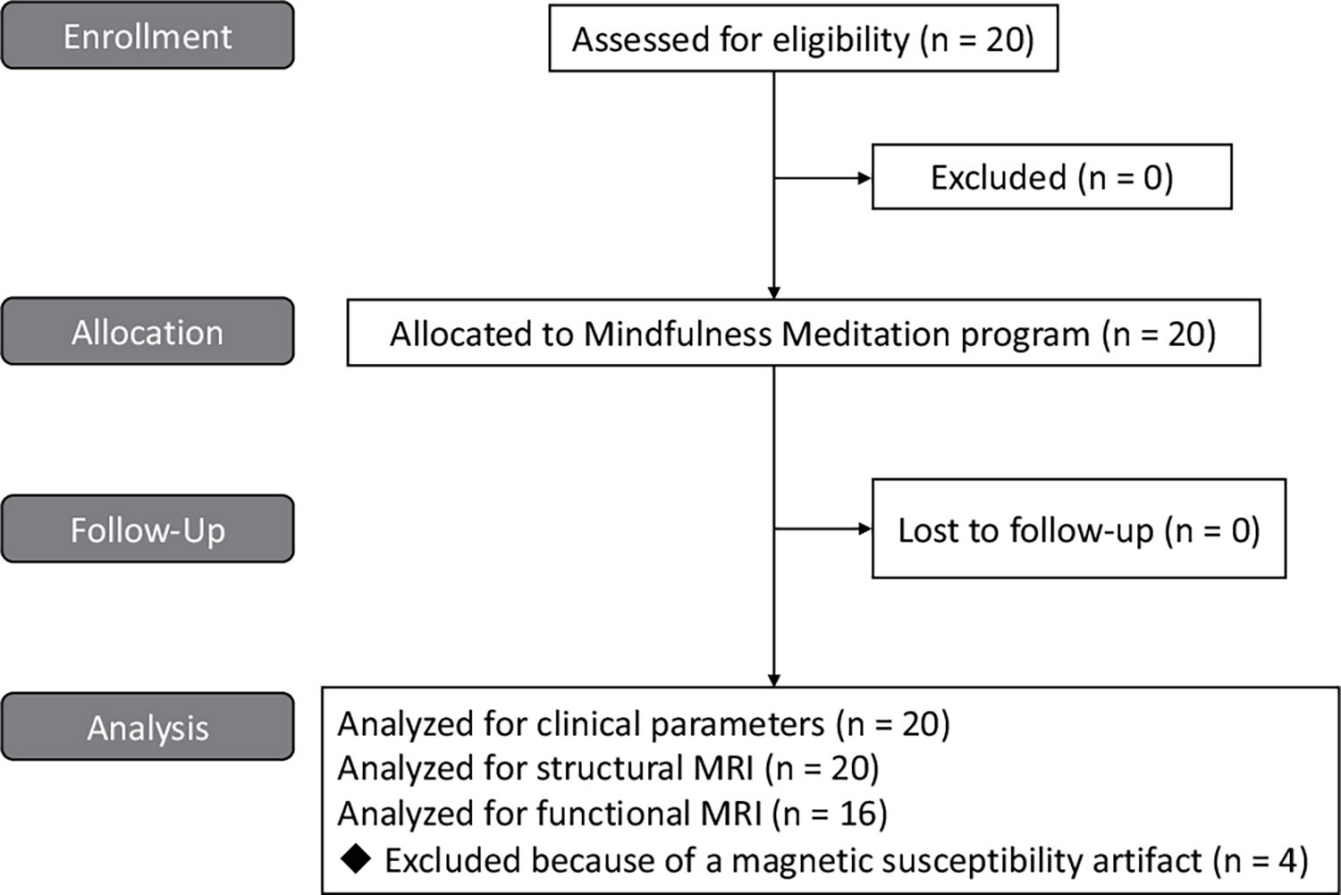
Flow diagram illustrating participant enrollment, allocation, and analysis.

**Fig 2:**
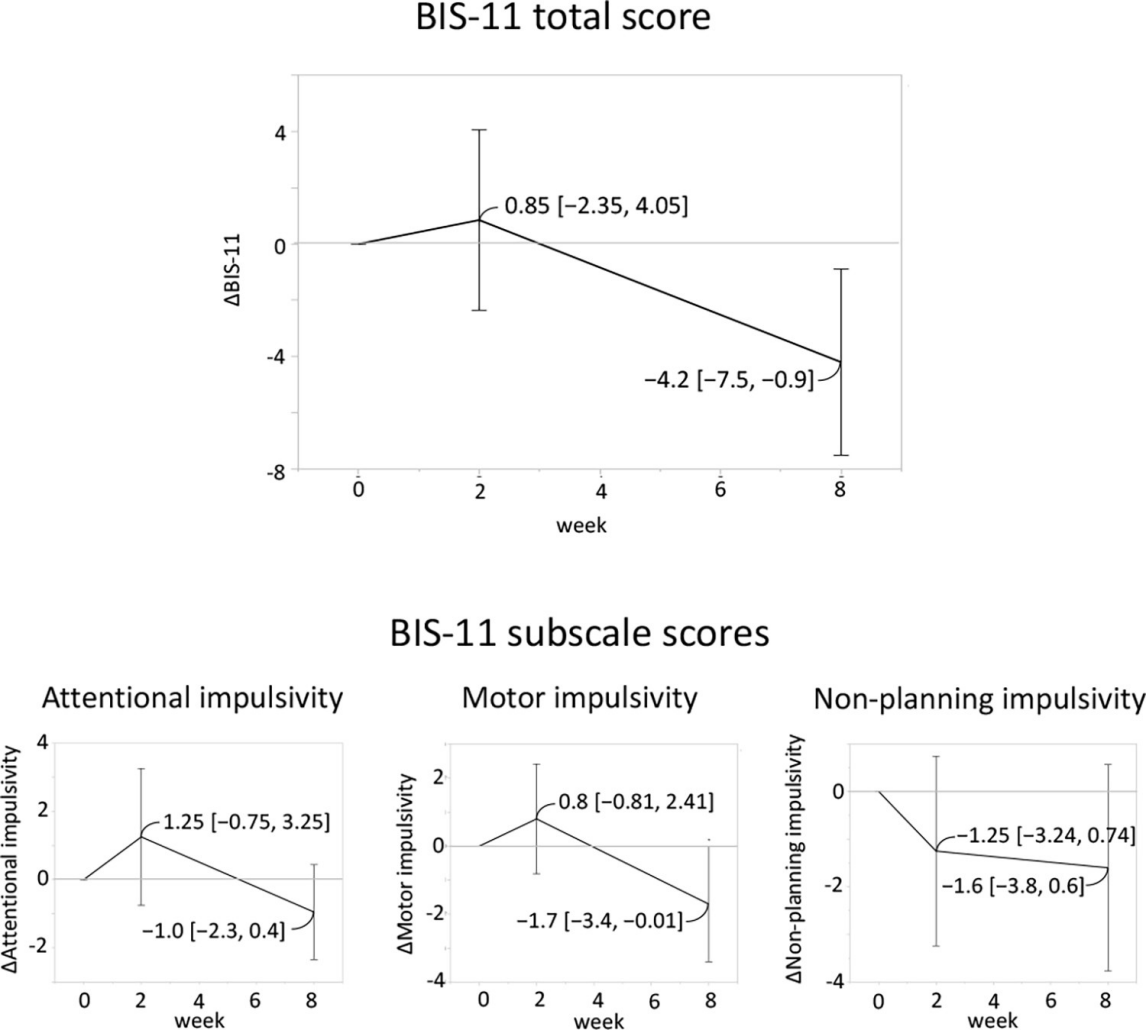
Primary outcome caused by an 8-week mindfulness meditation program. The BIS-11 total score was decreased at T2 compared to T0. Among the subscale scores, the motor impulsivity score was also decreased at T2, but not attention or non-planning impulsivity.

**Fig 3:**
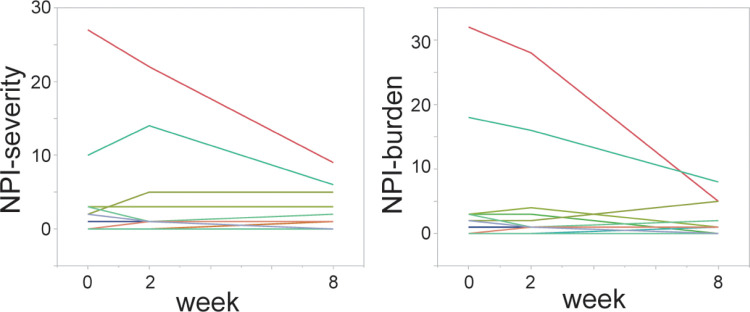
NPI-Q scores of 20 PD patients at T0, T1, and T2. The severity and burden scores remained low for most participants, but both scores were remarkably reduced for the two participants with relatively high baseline scores.

**Fig 4:**
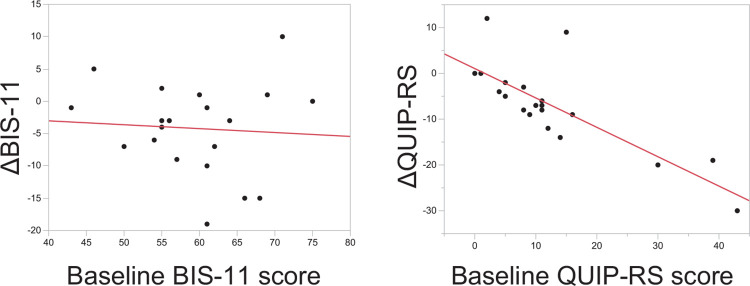
Scatter plot showing the relationship between baseline data and changes after mindfulness meditation program (BIS-11 and QUIP-RS).

All 20 patients with PD completed an 8-week mindfulness meditation program (Fig 1). The BIS-11 score was decreased from 59.5, [55.6, 63.3] (mean, [95% CI]) at T0 to 55.2, [50.3, 60.1] at T2 (Fig 2). Among the subscale scores of BIS-11, the decrease in motor impulsivity was the most remarkable (Fig 2). The other secondary outcomes are shown in Table 2 and Fig 3. No changes were observed between T1 and T0. The QUIP-RS and HADS anxiety scores also decreased, and the MAAS score increased at T2. The HADS depression score and PDQ-8 score showed a downward trend. The UPDRS motor score showed no change between T0 and T2. Since NPI-Q severity and burden scores at T0 were low (median [interquartile range], 0.5 [0, 3] and 0 [0, 3], respectively), mindfulness intervention did not result in a change in the mean score. However, in the two participants that showed high values at T0, the severity and burden scores were markedly decreased, and one participant had an apparent improvement in abnormal behavior associated with hypersexuality. There was no correlation between baseline BIS-11 and ΔBIS-11 at T2 (r = -0.070, 95% CI [-0.50, 0.38]). Conversely, the baseline QUIP-RS score had a strong correlation with ΔQUIP-RS (r = -0.80, [-0.91, -0.54]) (Fig 4). No adverse events were observed in this study.

**Table 2:** Secondary outcomes of clinical parameters.

	T0 (baseline)	T1 (week 2)	T2 (week 8)	Difference between T2 and T0	
				Mean (95% CI)	P-value
**QUIP−RS**	12.7 ± 11.7	9.9 ± 9.4	5.6 ± 7.1	-7.1 (-11.5, -2.7)	0.0033
**HADS_Depression**	5.8 ± 3.7	6.5 ± 3.9	4.4 ± 3.2	-1.4 (-3.1, 0.3)	0.11
**HADS_Anxiety**	6.3 ± 3.1	6.4 ± 3.4	4.4 ± 3.6	-1.9 (-3.1, -0.7)	0.0047
**PDQ−8 SI**	19.8 ± 15.0	23.6 ± 17.5	16.6 ± 10.7	-3.3 (-7.7, 1.2)	0.14
**MAAS**	71 ± 12.3	65.6 ± 12.2	76.3 ± 9.9	5.3 (0.7, 9.9)	0.026
**UPDRS part3**	16.8 ± 7.4	NA	16.8 ± 9.7	0 (-4.0, 4.0)	1.00

Data are presented as the mean ± standard deviation. The difference between T2 and T0 is presented as mean (95% CI) and P-value (one sample *t*-test).

CI, confidence interval; HADS, Hospital Anxiety and Depression Scale; MAAS, Mindful Attention Awareness Scale; PDQ-8 SI, Parkinson’s Disease Questionnaire-8 Summary Index; QUIP-RS, Questionnaire for Impulsive-Compulsive Disorders in Parkinson’s Disease–Rating Scale; UPDRS, Unified Parkinson’s Disease Rating Scale.

### Structural MRI and resting-state fMRI (rs-fMRI)

**Fig 5:**
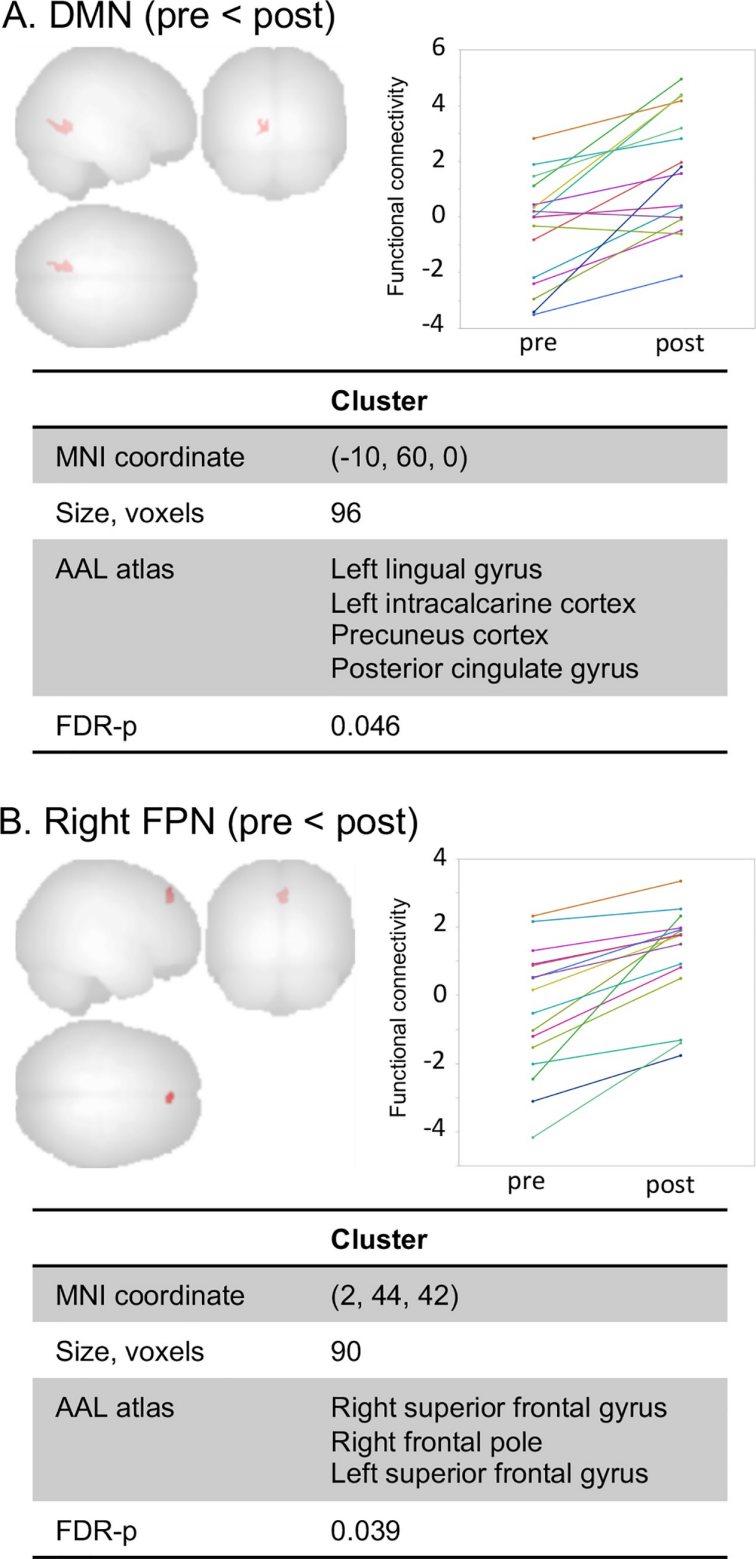
Brain regions whose functional connectivity was changed significantly due to mindfulness meditation. Mindfulness meditation increased the functional connectivity of the medial occipital lobe in the DMN (A) and medial frontal lobe in the right FPN (B). AAL, automated anatomical labeling; DMN, default mode network; FDR, false discovery rate; MNI, Montreal Neurological Institute.

Structural MRI was performed on all 20 patients, while rs-fMRI analysis was performed on 16 patients (Fig 1). Structural MRI analysis showed no significant difference between T0 and T2. The DMN, SN, and FPN were successfully identified by group ICA (S2 Fig). Between T0 and T2, a significant spatial property change was observed in the DMN and right FPN (Fig 5), but not in the left FPN or SN. Increased functional connectivity in the DMN was observed in a cluster including the precuneus, posterior cingulate gyrus, and left posterior lobe (FDR-p = 0.046), and increased functional connectivity in the right FPN was observed in the bilateral superior frontal gyrus (SFG) (FDR-p = 0.039).

## Discussion

This is the first report to investigate the effect of mindfulness meditation on controlling the impulsivity of patients with PD. In a brief 8-week mindfulness program, the BIS-11 scores decreased as mindfulness increased. Participants in this study had a relatively short disease duration of 5 years on average, had good motor and cognitive functions, and were receiving low to moderate doses of dopamine agonists. The BIS-11 score observed here was similar to that reported previously, which is a little higher than general population [7,32,44]. Thus, it is suggested that mindfulness meditation can reduce the slightly higher impulsivity of general PD patients closer to the level observed in the general population.

Impulsivity is multifaceted and involves various forms of response inhibition, reward evaluation, motivation, and cognitive control [45]. Recently, impulsivity has been divided into “motor” and “cognitive” impulsivity according to differences in pathophysiology, which also applies to PD [46]. Motor impulsivity is the tendency to “act without thinking,” while cognitive impulsivity is a more complex process that is the result of the suppression of previously activated cognitive contents. Among the BIS-11 subscale scores, attentional and non-planning impulsivity are both regarded as cognitive impulsivity. Our results suggested that mindfulness meditation was more effective at suppressing motor impulsivity than at suppressing cognitive impulsivity. However, this difference might be due to the small number of cases or a tendency to exert short-term effects on motor impulsivity rather than on cognitive impulsivity. This should be investigated in long-term controlled trials with a larger number of cases.

In this study, we included participants regardless of whether or not they met the diagnostic criteria for ICDs, because the main objective was to investigate the effect of controlling impulsivity in general PD patients. Conversely, it was recently pointed out that ICDs have a broad spectrum of severity and intensity [47]. Therefore, we used QUIP-RS as a secondary outcome to investigate the severity of ICDs, and found a decrease of QUIP-RS score following a brief program of mindfulness meditation. Furthermore, the higher the baseline QUIP-RS score, the higher the effect of the program. Thus, the QUIP-RS is a potential tool to measure the effects of mindfulness meditation in the future trials. When targeting cases with a cutoff QUIP-RS score of 6 points or more, which is the cutoff value of the Japanese version of QUIP-RS [43], a sample size of approximately 40 individuals is required when calculated with a significance level of α = 0.05 and power 1-β = 0.90. Conversely, when the BIS-11 score is used, a sample size of approximately 200 cases is estimated to be required.

The present study also suggested a decrease in the HADS anxiety score, but not in the depression score. A previous randomized trial showed that mindfulness yoga improved the HADS anxiety and depression scale scores [22]. One of the reasons that we did not observe a significant improvement in the depression scale score may be the low depression score at baseline. Furthermore, the amount of decrease in anxiety and depression after the intervention was smaller than in this previous study (in the present study, anxiety from 6.3 ± 3.1 to 4.4 ± 3.6, depression from 5.8 ± 3.7 to 4.4 ± 3.2; Kwok et al. [22], anxiety from 6.3 ± 3.6 to 3.0 ± 3.1, depression from 6.7 ± 3.4 to 3.5 ± 2.8), because of the short period of the intervention. The PDQ-8 score also showed an improving trend, but it was not significant. Our program did not involve exercise, so motor symptoms did not change, as expected. As most of the participants did not show burdensome neuropsychiatric symptoms, we could not show a significant decrease in the NPI-Q score. However, two participants with neuropsychiatric symptoms showed a marked decrease in NPI-Q scores, and so mindfulness meditation may contribute to an improvement of neuropsychiatric symptoms. In order to show the effect of brief mindfulness meditation on the above clinical symptoms, it is necessary to plan an appropriate study for each.

This is also the first report to investigate the effect of mindfulness meditation on the functional connectivity of patients with PD, and showed changes in the spatial properties of the DMN and right FPN. Several studies have revealed that mindfulness meditation can modulate the DMN [48]. Brewer et al. [18] reported that long-term meditators had lower DMN activity during meditation than non-meditators; however, blood oxygen level-dependent signal changes in the DMN decreased during meditation in the long-term meditators, while it increased in the non-meditators [18]. Thus, the change in functional connectivity in the DMN due to meditation is not linear and may change according to proficiency. Therefore, the increased functional connectivity of the DMN in the present study may have represented a short-term change in brain plasticity in subjects new to meditation.

The FPN is also an important network for mindfulness. The FPN is a key network in instantiating and flexibly modulating cognitive control [49]. A previous study also suggested that the functional connectivity of the FPN is increased by mindfulness training [50]. In the present study, functional connectivity was increased between the right FPN and bilateral SFG. The SFG is involved in self-awareness [51], has high functional connectivity with the DMN, and has direct connections with other prefrontal regions. Although the DMN and FPN have been considered to be two functionally anti-correlated networks, they interact and control attention [52]. Furthermore, the prefrontal cortex is activated by mindfulness meditation, particularly in the right hemisphere as well as the cingulate cortex [53]. Thus, the increased functional connectivity between the right FPN and SFG might be an important factor underlying the effect of mindfulness meditation.

There have been several reports on the relationship between impulsivity and large-scale brain networks, in particular the DMN, FPN, and SN [54–57], although the results vary depending on the methodology and subjects. We also reported altered functional connectivity in these three large-scale networks associated with the impulsivity of patients with PD [7]. Thus, the functional connectivity changes of the DMN and FPN associated with mindfulness meditation shown in the present study are in networks closely related to impulsivity. Therefore, these functional connectivity changes may have been associated with decreased impulsivity in patients with PD.

### Limitations

Since this study was a single-arm, open-label trial, it is necessary to be careful when interpreting its results. In particular, the main limitation is that we cannot distinguish the effect of mindfulness meditation from placebo effects, natural history or fluctuation of score in questionnaires. The prevalence of ICDs increases as the duration of PD or cumulative dose of dopamine agonists increases [58,59]. Although the natural history of impulsivity in PD is unclear, ICDs tend to be more severe as impulsivity increases [12]. Therefore, the severity of ICDs or impulsivity are unlikely to decrease spontaneously over a period of 8 weeks without changes to the drug regimen. Next, BIS-11 has a high test-retest reliability [31,32], and it is considered that a decrease of its score is not accidental or “regression to the mean”. Indeed, the BIS-11 score did not change at 2 weeks, as expected. Conversely, it is especially difficult to eliminate the placebo effect due to the methodology of mindfulness meditation. Well-designed double-blind randomized controlled trials are needed in the future. Second, impulsivity and severity of ICBs were only evaluated using the BIS-11 and QUIP-RS. If we utilized additional test, such as the Iowa gambling task or stop-signal reaction time, the data may become more robust. Third, this study assessed the short-term impact of an 8-week intervention, and did not examine its long-term effects. Fourth, although the rs-fMRI analysis used in this study is a commonly used approach, rs-fMRI analysis has many problems that remain to be solved and is still the subject of discussion. In particular, for movement disorder such as PD, involuntary movements can cause artifacts in rs-fMRI. This study targeted cases without problems with involuntary movements, which may be a selection bias. Furthermore, the results of this rs-fMRI analysis were exploratory and remain to be substantiated.

## Conclusions

This open-label, single-arm pilot study provided preliminary data for the use of mindfulness meditation to control the impulsivity of patients with PD. Our findings suggest that a brief mindfulness meditation program may be effective in controlling impulsivity in PD. Furthermore, it was suggested that mindfulness meditation changes the functional connectivity of the DMN and right FPN. Randomized controlled trials are needed in the future to verify these effects.

## Supporting information

S1 Fig Study design. (PDF)

S2 Fig The spatial properties of functional connectivity in the DMN, FPN, and SN at pre- (T0) and post- (T2) intervention. T0 and T2 showed large-scale networks with high reproducibility, but post-mindfulness meditation formed a broader network, especially in the precuneus and posterior cingulate gyri in the DMN. DMN, default mode network; FPN frontoparietal network; SN, salience network.
(PDF)

S1 File The study data. (XLSX)

S2 File Protocol Japanese. (DOCX)

S3 File Protocol English. (DOCX)

S4 File TREND statement checklist. (PDF)
