# Supplementary figures and images for: A single-arm open-label pilot study of brief mindfulness meditation to control impulsivity in Parkinson’s disease
Source: PLoS One. 2022 Apr 6;17(4):e0266354. doi: 10.1371/journal.pone.0266354 (PMC8985985; doi:10.1371/journal.pone.0266354)

Weekly group sessions

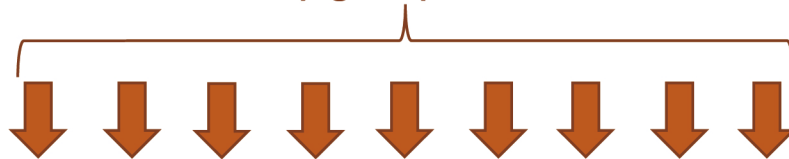

Daily Mindfulness Meditation

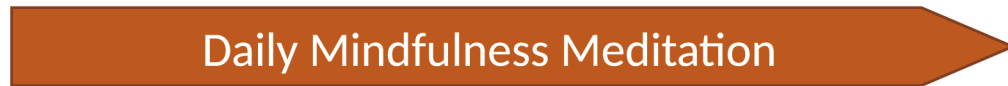

Screening

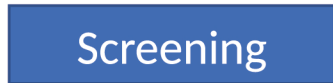

Assessment

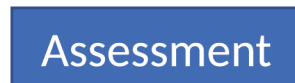

week

-10

0

2

8

10

T0

T1

T2

Supplement: S1 Fig — (PDF) [file pone.0266354.s001.pdf]

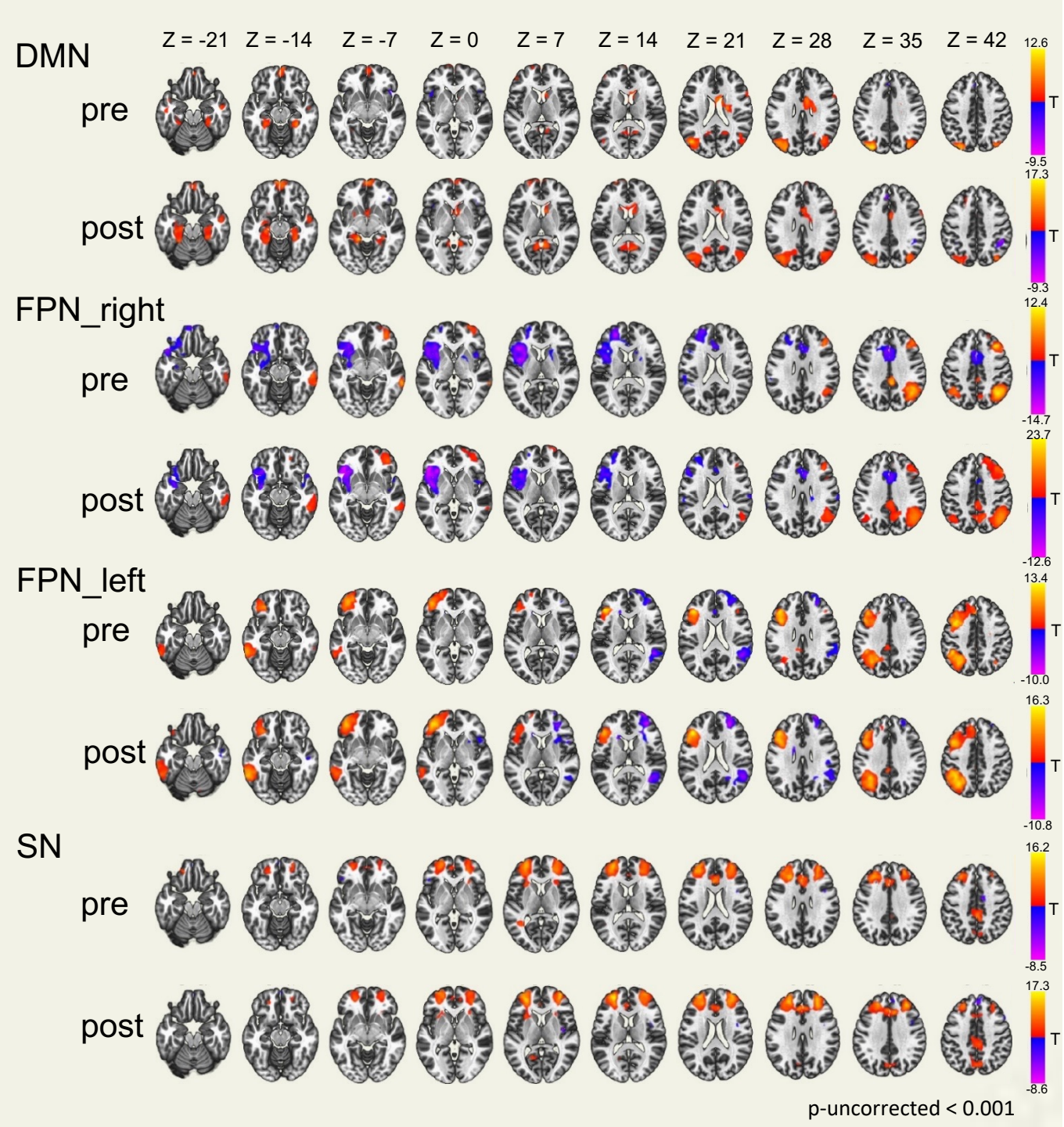

Supplement: S2 Fig — T0 and T2 showed large-scale networks with high reproducibility, but post-mindfulness meditation formed a broader network, especially in the precuneus and posterior cingulate gyri in the DMN. DMN, default mode network; FPN frontoparietal network; SN, salience network. (PDF) [file pone.0266354.s002.pdf]
