# Supplementary material for: A single-arm open-label pilot study of brief mindfulness meditation to control impulsivity in Parkinson’s disease
Source: PLoS One. 2022 Apr 6;17(4):e0266354. doi: 10.1371/journal.pone.0266354 (PMC8985985; doi:10.1371/journal.pone.0266354)
Supplement: S2 File — (DOCX) [file pone.0266354.s004.docx]

|  |
| --- |
| 研究計画書  **課題名**  科学的試験名：パーキンソン病の衝動性に対するマインドフルネス瞑想法の有効性に関する  単群、オープンラベル、パイロット試験  A pilot study of mindfulness meditation on trait impulsivity of Parkinson’s disease  (MIMP study)  一般向け試験名：パーキンソン病患者に対するマインドフルネス瞑想法の有効性研究 |
|  |
| **[研究責任者]**  髙　真守  　和歌山県立医科大学 脳神経内科  　和歌山県和歌山市紀三井寺８１１－１  　連絡先：073 - 441 - 0655  　　　　 jinsoo@wakayama-med.ac.jp  **[研究事務局]**  　髙　真守  　和歌山県立医科大学 脳神経内科  　和歌山県和歌山市紀三井寺８１１－１  　連絡先：073 - 441 - 0655  　　　　 jinsoo@wakayama-med.ac.jp |
| 第1.1版 |

変更履歴表

| 改訂番号 | 年月日 | 変更内容 | 変更理由 |
| --- | --- | --- | --- |
| 1.0 | 2019.06.18 | 制定 | - |
| 1.1 | 2019.10.23 | 5-3. 登録に関する注意事項  ⑤登録日から28日以内  →70日以内 | グループレッスンの期間が9週間であり、登録日から次回開催まで最長63日待つ必要があり、加えて観察期の検査に7日間の猶予をもたせるため |
|  |  | 9-2. 検査・報告スケジュール  観察期間：-28日以内  →-70日以内 | 上記と同様 |
|  |  |  |  |

**概 要**

| 項目 | 内容 |
| --- | --- |
| 目的 | パーキンソン病患者の衝動性亢進に対する、マインドフルネス瞑想の有効性を評価する。 |
| 研究対象者 | 当科通院中のパーキンソン病患者を対象とする。 |
| 選択基準 | - MDS clinical diagnostic criteria for Parkinson’s diseaseにおいてClinically Established PDもしくはClinically Probable PDに該当する症例 - 登録時に20歳以上の症例 - 本研究の概要を理解し、文書で同意を得られる症例 |
| 除外基準 | - 高度認知症もしくは幻覚妄想状態により、研究の遂行が困難な症例 - 高度難聴、高度視力障害により、研究の遂行が困難な症例 - 意識障害を認める症例 - 他の介入試験に参加中の症例 - 登録前28日以内に抗パーキンソン病薬が変更された症例 - 登録前84日以内に新規の抗精神病薬、抗うつ薬を開始された症例 - 登録前84日以内に症候性の脳卒中、脳炎を発症した症例 - 日常的に瞑想もしくはヨガを行っている症例 |
| 被験者の同意 | 担当医は試験の開始に先立ち，同意説明文書・同意書を用いて被験者にわかりやすく説明を行い，被験者には十分に考える時間を与え，被験者が試験の内容をよく理解したことを確認したもとで試験への参加について依頼する．同意文書は説明をした医師名，説明を受け同意した被験者名，同意を得た日付を記載し，医師，被験者各々が署名する．そして，1部コピーし，1部は被験者本人に手渡し，同意書の原本は試験実施機関で定められた保管場所に保管する． |
| 介入方法 | マインドフルネス瞑想  週に1回、専門の臨床心理士立ち会いの元に、適宜指示を受けながら行う。初回はオリエンテーションを含め、約30分を想定する。マインドフルネス瞑想は、ボディスキャン、呼吸法を含む。同様の15分間のマインドフルネス瞑想の自己練習を、週に6日以上行い、日誌に記載する。いずれも安静坐位もしくは臥位にて行うため侵襲度は低く，有害事象が起こる可能性は極めて低い．評価は各種アンケート用紙および担当医の診察にて行う．試験の前後で頭部MRI撮像を行う。 |
| 研究方法  （治療スケジュール等） | 当科にて加療中のパーキンソン病患者および健常対照者に対して、本研究の目的、方法を説明した上で研究参加を依頼する。文書にて同意を得て登録を行う。登録後、観察期に認知機能検査（MMSE）、運動症状評価（UPDRS part3、Hoehn-Yahr重症度分類）、頭部MRI撮像、各種質問紙評価（BIS-11、QUIP-RS-J、HADS、PDQ-8、MAAS，NPI-Q）を行う。ウェアリングオフ現象がある症例に関しては、MMSE、UPDRS、頭部MRIの撮像は、オン時に行う。閉所恐怖症など、MRI撮像が困難な場合はMRI撮像を行わないこととする。  　マインドフルネス瞑想は、週に1回、専門の臨床心理士立ち会いの元に、適宜指示を受けながら行う。初回はオリエンテーションを含め、約30分を想定する。マインドフルネス瞑想は15分間行う。マインドフルネス瞑想は、ボディスキャン、呼吸法を含む。同様の15分間のマインドフルネス瞑想の自己トレーニングを、週に6日以上行い、日誌に記載する。  　2週後、8週後（前後1週間を許容）に再度、観察期と同様に各種評価を行い、8週後の評価終了後、14日以内に頭部MRIを撮像し、試験を終了とする。評価項目、スケジュールは、「9．検査項目及び報告すべき情報」を参照とする。 |
| 併用禁止薬剤及び  併用禁止療法 | 登録日より観察期間終了日までは、抗パーキンソン病薬，抗精神病薬、抗うつ薬の新規追加および変更を禁止する。 |
| 観察・検査スケジュールの概略 | ・患者背景として、年齢、性別、身長、体重、病歴（パーキンソン病罹病期間、発症年齢など）、内服薬、既往歴、合併症を聴取し、認知機能（MMSE）を評価する。  ・運動症状の評価として、MDS-UPDRS、Hoehn-Yahr重症度分類を評価する。  ・質問紙として、衝動性評価尺度（BIS-11）、衝動制御障害尺度（QUIP-RS-J）、抑うつおよびアパシー評価尺度（HADS）、QOL評価尺度（PDQ-8）、マインドフルネス評価尺度（MAAS）を、観察期間、2週後、8週後に行う。介護者がいる場合は，精神症状評価としてNPI-Qも行う．  ・頭部MRI撮像を観察期間、試験終了後14日以内に行う。 |
| 主要評価項目 | 8週間のマインドフルネス瞑想によって、衝動性評価尺度Barratt impulsiveness scale 11th version(BIS-11)が有意に低下するかどうかを検証する。 |
| 目標症例数 | 目標登録被験者数： 20例 |
| 被験者登録期間 | 学長による研究実施許可日（＝承認日）～ 2021年8月31日 |
| 研究実施期間 | 学長による研究実施許可日（＝承認日）～ 2024年8月31日 |

# 1. 研究目的及び意義

## 1-1. 目的

　パーキンソン病患者の衝動性亢進に対する、マインドフルネス瞑想の有効性を評価する。

## 1-2. 本研究の意義

　本研究を行うことによって，マインドフルネス瞑想がパーキンソン病の衝動性亢進をどの程度抑えることができるかについてのデータを得ることができる．このパイロット研究の結果を用いて，第三相ランダム化比較試験へつなげることを想定する．

# 2. 背景

　　パーキンソン病は，10万人に100〜150人程度が罹患する、アルツハイマー病に次いで多い神経変性疾患である。その本態はドパミン神経の変性脱落であり、それによって動作緩慢、振戦、筋強剛などの運動症状を来す。未だ根治的治療法は存在せず，治療法の主体は脳内ドパミン濃度低下に伴う諸症状を改善するためのドパミン補充療法であり、levodopa製剤、ドパミンアゴニストなどがパーキンソン病診療ガイドラインにおいても推奨されている。一方で，パーキンソン病の運動症状は疾患の一側面に過ぎないことが指摘されてきており、非運動症状への対応も重要視されてきている。抑うつやアパシー、アンヘドニア、衝動制御障害（Impulse control disorders: ICDs）などの精神症状もQOLを低下させ得る大きな問題である。ICDsはパーキンソン病患者の13%程度が経験すると報告されており(Weintraub, 2010 ^17^)、病的賭博、買いあさり、むちゃ食い、性行動異常などが含まれる．またICD関連症状として、punding（単純な行為を意味もなく繰り返すこと）やドパミン調節障害症候群（必要以上にドパミン補充療法を渇望すること）なども挙げられる。その対応としては、ドパミン補充療法の減量が一つの方法であるが、それによって運動症状の増悪や離脱症状（dopamine agonist withdrawal syndrome: DAWS）が起こることがあり，対応に苦慮することが多い。ICDsを直接的に改善させるための薬物治療のエビデンスは未だ乏しいのが現状である．

　パーキンソン病のICDsの病態機序については不明な点が多い．前述のように，パーキンソン病では，黒質緻密部のドパミン神経脱落により運動症状が出現するが，報酬や目標志向型の行動に中心的な役割を担っている腹側被蓋野は比較的保たれている．そこへ運動症状改善のためのドパミン補充療法を行うことによって，腹側被蓋野から腹側線条体や前頭葉への過剰興奮が起こり，ICDsを起こすことが想定されている(Weintraub, 2008 ^16^)．ただしICDsを来たしやすい症例とそうでない症例があり，ICDsを認める患者では報酬系を含めた何らかの脆弱性が存在することも想定されている．さらに，元々の衝動性が高い性格傾向の症例において、ICDsの重症度がより高いという報告もある(Marin-Lahoz, 2018 ^8^)．我々の先行研究においても、パーキンソン病患者は健常対象者に比べて、有意に衝動性が高いことが示されており、衝動性亢進そのものが，これまで見逃されていたパーキンソン病による精神症状である可能性がある．

　近年，精神医学領域において非薬物療法としてマインドフルネス瞑想が注目されてきている．そのきっかけとなったのは，Jon Kabat-Zinnらが開発したマインドフルネスストレス低減法 Mindfulness based stress reduction (MBSR)であり，これは、仏教の瞑想法から宗教色を取り除いた，8週間のトレーニングプログラムとして開発されたものである。そこから，毎日15分程度の簡易化したプログラムやマインドフルネス認知療法 mindfulness-based cognitive therapy (MBCT)などが派生しており，これらの治療によって，うつ病や不安障害，薬物依存などを有意に改善させることや，うつ病の再発予防効果など，多くのエビデンスが集積されてきている．(Khoury, 2013 ^6^;Piet, 2011 ^10^;Strauss, 2014 ^14^;Gilmartin, 2017 ^5^)．さらに最近では注意欠陥多動性障害の症状改善(Xue, 2019 ^19^)，薬物依存の改善(Wilson, 2017 ^18^)、中高生における衝動性低下(Franco, 2016 ^4^)などの報告もある．このように、抑うつや薬物依存、衝動性などの精神症状に対して、マインドフルネス瞑想は確立した非薬物療法となってきている。一方で近年，パーキンソン病患者においても抑うつに対するMBSRの効果が第三相試験にて証明されている (McLean, 2017 ^9^;Kwok, 2019 ^7^)．このような背景から、パーキンソン病のICDsやICD関連症状、さらに衝動性の亢進に対して、マインドフルネス瞑想は有効性が期待できる非薬物療法であるが、その有効性を証明した報告はない。そのため，今回我々は、パーキンソン病の衝動性亢進が、マインドフルネス瞑想によって改善するかどうかを調べるために、パイロット試験として、単アームの介入試験を計画した。

　さらに、衝動性亢進の病態生理、マインドフルネスの脳機能にもたらす変化を調べるために、頭部MRIによる構造および機能画像の評価を併せて行う。生体での脳機能障害を測定する方法は多くないが，近年、安静時機能的MRIによって非侵襲的に脳の機能的結合性を測定する方法が開発されてきている．安静時機能的MRIを用いて，安静時に賦活化されるdefault mode networkと衝動性の関連が指摘されており(Zhao, 2017 ^21^)、さらにマインドフルネス尺度（MAAS）が注意に関連したネットワークや、大規模ネットワーク間の結合性と関連があること(Bilevicius, 2018 ^1^)、MBSRによってdefault mode networkの過剰興奮が抑制されるという報告(Brewer, 2011 ^2^)などがある。パーキンソン病においても，黒質線条体系の障害のため，線条体と運動系の機能的結合性が低下すること，またドパミン補充療法にてその機能的結合性が改善することが示されている(Szewczyk-Krolikowski, 2014 ^15^)．ICDsに関しては，報酬系を司る腹側線条体での機能障害が指摘されているが，機能低下を示唆する報告もあれば，過剰興奮を示唆する報告もある．さらに報酬系以外のassociation striatumと側頭葉下面との機能的結合性低下を示唆する報告もあり(Carriere, 2015 ^3^)，より広範な脳機能異常が示唆されてきている．さらに機能異常のみでなく，パーキンソン病患者においてMBSRが扁桃体や海馬などの灰白質密度を増加させることも報告されている(McLean, 2017 ^9^;Kwok, 2019 ^7^)．このような背景の中，今回我々は副次的探索的項目として、パーキンソン病に対してマインドフルネス瞑想前後において、安静時機能的MRIおよび構造画像を撮像することによって、その構造上および機能的変化がどのように起きるかについて、過去の報告との類似点および相違点を検討する。

# 3. 研究計画

## 3-1. 研究対象集団

　当科通院中のパーキンソン病患者を対象とする。

## 3-2.　試験デザイン

(1) 試験の種類

　単施設によるパイロット試験

(2) 試験群

　試験治療：マインドフルネス瞑想

(3) 試験デザインの説明

　本試験は．パーキンソン病患者の衝動性に対するマインドフルネス瞑想に関する有効性を探索するためのパイロット研究である．

## 3-3. 研究期間

　研究期間：2019年9月～2024年8月 (研究期間：5年)

　登録期間：2019年9月1日～2021年8月31日

## 3-4. 目標登録症例数

　目標登録症例数はパーキンソン病患者20例

## 3-5. マインドフルネス瞑想および検査法の概要

　3-5-1. マインドフルネス瞑想および臨床評価

マインドフルネス瞑想の方法は、MBSRに準じるが，15分間の短縮バージョンで行う(Gilmartin, 2017 ^5^)．週に1回、専門の臨床心理士1名が立ち会いの元に、適宜指示を受けながら行う。初回はオリエンテーションを含め、約30分を想定する。マインドフルネス瞑想は、ボディスキャン、呼吸法を含む。同様の15分間のマインドフルネス瞑想の自己練習を、週に6日以上行い、日誌に記載する。いずれも安静坐位もしくは臥位にて行うため侵襲度は低く，有害事象が起こる可能性は極めて低い．評価は後述のアンケート用紙および担当医の診察にて行う．頭部MRI以外については通常の診療の範囲内で行う．

　3-5-2. 頭部MRI

　頭部MRIは，和歌山南放射線科クリニックにて行う．頭部単純MRI（T1強調画像，T2強調画像，安静時機能的MRI）を撮像し，撮像時間は約30分である．通常診療と同様の手順で行うが，保険適応外であるため，経費に関しては研究費を用いる．観察期と8週目の最終評価日以後14日以内の2回行う．

## 3-7. 本研究参加に伴って予想される利益

　　本研究に参加することによって、衝動制御障害および衝動性の改善を認める可能性がある。さらに、パーキンソン病患者で併存する抑うつ症状が改善する可能性もある。その他、集中力の向上など、予期せぬ健康上の利益をもたらす可能性もある。非侵襲的であり、副作用を認める可能性は極めて少ない。

## 3-8. 本研究参加に伴って予想されるリスクと不利益

　　本研究参加に伴って、毎週15分のグループレッスン、自宅での毎日の15分間の自己練習が課せられる。通院費は自己負担となる。研究期間中には薬剤の変更が制限される。マインドフルネス瞑想法による身体的な負担はごく軽微であるが、15分間の坐位保持が困難な参加者に対しては、臥位やその他の姿勢で行うことも可能とする。

# 4. 適格基準

## 4-1 選択基準

- MDS clinical diagnostic criteria for Parkinson’s diseaseにおいてClinically Established PDもしくはClinically Probable PDに該当する症例
- 登録時に20歳以上の症例
- 本研究の概要を理解し、文書で同意を得られる症例

## 4-2 除外基準

- 高度認知症もしくは幻覚妄想状態により、研究の遂行が困難な症例
- 高度難聴、高度視力障害により、研究の遂行が困難な症例
- 意識障害を認める症例
- 他の介入試験に参加中の症例
- 登録前28日以内に抗パーキンソン病薬が変更された症例
- 登録前84日以内に新規の抗精神病薬、抗うつ薬を開始された症例
- 登録前84日以内に症候性の脳卒中、脳炎を発症した症例
- 日常的に瞑想もしくはヨガを行っている症例

# 5. 登録

## 5-1. 症例の登録の手順

　対象患者が選択基準をすべて満たし，除外基準のいずれにも該当しないことを試験担当医師が確認し，必要事項を「症例登録票」に記載し、ファイルに保管する。試験担当医師は，登録完了後に治療を開始する．

## 5-2. 症例登録の問い合わせ先

　登録に関する意見・質問等は，登録事務局に問い合わせることとする．

施設名：和歌山県立医科大学　脳神経内科

　　　　 〒640-8510 和歌山市紀三井寺811-1

　　　　　　Tel: 073-441-0655

　　　　　　Fax: 073-441-0655

　　　　　　E-mail: jinsoo@wakayama-med.ac.jp

## 5-3. 登録に関する注意事項

① プロトコール治療開始後の登録はいずれの場合も許容されない．

② 記載方法に関して疑問がある場合は，登録事務局に問い合わせること．

③ データの研究利用の拒否を含めた同意撤回があった場合を除いて，一度登録された被験者は登録取り消し(データベースから抹消)されない．重複登録の場合は，いかなる場合も初回の登録情報(登録番号，割付群)を採用する．

④ 誤登録や重複登録が判明した際には，速やかに登録事務局に連絡すること．

⑤ 登録日から70日以内(同一曜日は可)に試験治療を開始すること．

# 6. 研究の実施

## 6-1 研究の流れ

　当科にて加療中のパーキンソン病患者および健常対照者に対して、本研究の目的、方法を説明した上で研究参加を依頼する。文書にて同意を得て登録を行う。パーキンソン病患者に対しては、登録後、観察期に認知機能検査（MMSE）、運動症状評価（UPDRS part3、Hoehn-Yahr重症度分類）、頭部MRI撮像、各種質問紙評価（BIS-11、QUIP-RS-J、HADS、PDQ-8、MAAS，NPI-Q）を行う。ウェアリングオフ現象がある症例に関しては、MMSE、UPDRS、頭部MRIの撮像は、オン時に行う。閉所恐怖症など、MRI撮像が困難な場合はMRI撮像を行わないこととする。

　マインドフルネス瞑想は、週に1回、専門の臨床心理士立ち会いの元に、適宜指示を受けながら行う。初回はオリエンテーションを含め、約30分を想定する。マインドフルネス瞑想は15分間行う。マインドフルネス瞑想は、ボディスキャン、呼吸法を含む。同様の15分間のマインドフルネス瞑想の自己トレーニングを、週に6日以上行い、日誌に記載する。

　2週後、8週後（前後1週間を許容）に再度、観察期と同様に各種評価を行い、8週後の評価終了後、14日以内に頭部MRIを撮像し、試験を終了とする。最終のMRI撮像日までマインドフルネス瞑想の自己トレーニングは継続する。評価項目、スケジュールは、「9．検査項目及び報告すべき情報」を参照とする。

併用禁止薬

登録日より観察期間終了日までは、抗パーキンソン病薬，抗精神病薬、抗うつ薬の新規追加および変更を禁止する。

## 6-2 プロトコール治療の中止・完了基準

　次のいずれかの項目に該当するときは当該被験者に対する調査（評価・観察）を中止する。

1. 医師判断

- 原疾患が悪化し中止すべきと判断された場合
- 有害事象が発現し中止すべきと判断された場合
- その他、本研究の対象として継続不可能と判断された場合

1. 被験者が同意を撤回した場合
2. 研究計画書の不遵守

適格基準を満たさないことが判明した場合（適格基準を満たしていなかったことが登録後に判明した場合、中止とみなさず「登録後不適格」とし、研究参加を終了する）

1. その他

被験者が死亡した場合

# 7. 有害事象

　有害事象とは，プロトコール治療との因果関係を問わず，当該プロトコール治療終了1週後までに被験者に生じた全ての好ましくない又は意図しない疾病又は障害並びにその兆候をいう．本試験前に存在していた症状の臨床的に有意な悪化もまた有害事象である．頻度や程度に於いて臨床的意義がないと考えられる生理的変動は有害事象として考えない．被験者に有害事象を認めた場合は，試験担当医師は直ちに被験者の安全性の確保および適切な処置を行うとともに，その内容について症例報告書に記載する．

## 7-1 基本的事項

被験者の安全性を確保するために，研究責任者および研究分担者は，以下の基本的事項を遵守する．

1) 研究責任者又は研究分担者は，被験者の選択基準および除外基準を遵守する．

2) 被験者が本試験の研究責任者および研究分担者以外の医師の治療を受ける場合には，本試験に参加していること，および本試験の内容を当該医師に通知する．

3) 本試験完了・中止時に継続している有害事象については，消失もしくは軽快するまで，可能な限り追跡調査を行う．

4) 被験者が健康状態の異常を感じた場合には，直ちに研究責任者又は試験担当医師に連絡するよう指導する．

5) 研究責任者および研究分担者は，被験者に有害事象が生じ，治療が必要であると認められるときは，その旨を当該被験者に伝え，適切な医療を提供する．

## 7-2 予期される有害事象

　予期される有害事象はとくにない．

## 7-3 重篤な有害事象

重篤な有害事象とは，有害事象のうち以下のいずれかに該当するものをいう．

1. 死に至るもの
2. 生命を脅かすもの
3. 治療のための入院または入院期間の延長が必要となるもの
4. 永続的又は顕著な障害・機能不全に陥るもの
5. 子孫に先天異常を来すもの

## 7-4 有害事象の報告

　重篤な有害事象への対応は，和歌山県立医科大学 倫理審査委員会「人を対象とする医学系研究における重篤な有害事象発生時の対応手順書」を遵守する．研究責任者は，試験薬との因果関係を問わず適切な処置を行うと共に，直ちに学長にその内容を文書で報告する．予期されない重篤な有害事象が発生し,当該研究との直接の因果関係が否定できない場合には，学長が速やかに厚生労働省に報告を行う．

# 8. エンドポイントの定義

## 8-1. 主要評価項目

　8週間のマインドフルネス瞑想によって、衝動性評価尺度Barratt impulsiveness scale 11th version(BIS-11)が有意に低下するかどうかを検証する。

## 8−2．副次的評価項目

1. 臨床症状に対する質問紙評価

いずれも観察期間、2週後、8週後に行う。

1. 衝動性評価尺度：BIS-11
2. 衝動制御障害評価尺度：QUIP-RS-J
3. パーキンソン病のQOL尺度：PDQ-8
4. マインドフルネス尺度：MAAS
5. 抑うつおよびアパシーの評価：HADS
6. 精神症状および介護負担度の評価：NPI-Q
7. パーキンソン病の運動症状評価

いずれも観察期間、8週後に行う。

1. MDS-UPDRS part3
2. Hoehn-Yahrスコア
3. 認知機能評価

観察期に行う

1. Mini-Mental State Examination (MMSE)
2. MRIによる探索的評価

観察期および8週後に行う。

1. 脳機能の測定：安静時機能的MRI
2. 脳萎縮の評価：Voxel-based morphometry
3. T1w/T2w ratio image

# 9. 検査項目とスケジュール

## 9-1. 検査項目及び報告すべき情報

- 患者背景として、年齢、性別、身長、体重、病歴（パーキンソン病罹病期間、発症年齢など）、内服薬、既往歴、合併症を聴取し、認知機能（MMSE）を評価する。
- 運動症状の評価として、MDS-UPDRS、Hoehn-Yahr重症度分類を評価する。
- 質問紙として、衝動性評価尺度（BIS-11）、衝動制御障害尺度（QUIP-RS-J）、抑うつおよびアパシー評価尺度（HADS）、QOL評価尺度（PDQ-8）、マインドフルネス評価尺度（MAAS）を、観察期間、2週後、8週後に行う。介護者がいる場合は，精神症状評価としてNPI-Qも行う．
- 頭部MRI撮像を観察期間、試験終了後14日以内に行う。
- 日誌を用いたマインドフルネス瞑想の達成率評価（15分、週に6日以上）

## 9-2. 検査・報告スケジュール

|  | 観察期間 | 介入開始日 | 介入期間 | | 中止・終了時 |
| --- | --- | --- | --- | --- | --- |
| 時期 | -70日以内 | 0週 | 2週後 | 8週後 |  |
| 登録 |  |  |  |  |  |
| 適格性評価 | ○ |  |  |  |  |
| 同意取得 | ○ |  |  |  |  |
| MMSE | ○ |  |  |  |  |
| 介入 |  |  |  |  |  |
| マインドフルネス瞑想 |  |  |  |  |  |
| 評価 |  |  |  |  |  |
| BIS-11 | ○ |  | ○ | ○ |  |
| QUIP-RS-J | ○ |  | ○ | ○ |  |
| HADS | ○ |  | ○ | ○ |  |
| PDQ-8 | ○ |  | ○ | ○ |  |
| MAAS | ○ |  | ○ | ○ |  |
| Hoehn-Yahr | ○ |  |  | ○ |  |
| MDS-UPDRS part3 | ○ |  |  | ○ |  |
| NPI-Q | ○ |  | ○ | ○ |  |
| MRI | ○ |  |  |  | ○ |
| 日誌評価 |  |  | ○ | ○ |  |
| 有害事象 |  |  |  |  |  |

# 10. 統計的事項

## 10-1. 解析対象集団

　本試験における解析対象集団の定義は以下のとおりとする．必要に応じて、データ固定前に研究責任者，研究事務局及びデータマネジメント責任者が協議を行い決定する．

　登録された患者のうち，重複登録や誤登録を除いた症例を「全登録例」とする．全登録例から「不適格例」を除く集団を「全適格例」とする．

　・有効性解析対象集団

　　全適格例のうち，登録後に本試験の適格性基準を満たしていないことが判明した症例を除いたプロトコール治療が一度でも施行された症例．

　・安全性解析対象集団

　　全登録例のうち，プロトコール治療の一部または全部を受けた症例．

## 10-2. 目標登録症例数の設定根拠

　パーキンソン病の衝動性に対するマインドフルネス瞑想の効果を調べた報告はない．若年者の衝動性に対するマインドフルネストレーニングの報告では、介入前のBIS-11スコアが82.49±10.27であったものが、介入後には71.2±9.16に低下したと報告されている。我々の先行研究において，パーキンソン病患者のBIS-11スコアは62.6±8.7であり，健常対象者は56.6±10.6であった．マインドフルネス瞑想法によりBIS-11の変化量が-10.0±8.0、臨床的に意義のある最小の変化量を-5.0と仮定する。有意水準両側10％、検出力80%の場合、サンプルサイズは18例必要である．若干の不適格例などを考慮して20例とした．和歌山県立医科大学附属病院 脳神経内科において，通院中のパーキンソン病の患者は，年間300例程度であり，目標症例数の20例は充分エントリー可能と考える．必要症例数の算出にはSAS9.4を使用した。

## 10-3. 主要評価項目の評価

　8週後のBIS-11変化量の両側95%信頼区間及びt検定のp値を算出する。また、各時点の実測値及び変化量の要約統計量を算出する。

## 10-4. 副次的評価項目の評価

　各評価項目に対して、各時点の実測値及び変化量の要約統計量を算出する。

# 11. 倫理的事項

## 11-1. 遵守すべき諸規則

　本研究(試験)に関連するすべての研究者は「ヘルシンキ宣言(2013年10月 フォルタレザ改訂版)」(日本医師会訳)および「人を対象とする医学系研究に関する倫理指針(平成29年5月30日施行)」に従って本研究を実施する．

## 11-2. インフォームド・コンセント

　担当医は試験の開始に先立ち，同意説明文書・同意書を用いて被験者にわかりやすく説明を行い，被験者には十分に考える時間を与え，被験者が試験の内容をよく理解したことを確認したもとで試験への参加について依頼する．同意文書は説明をした医師名，説明を受け同意した被験者名，同意を得た日付を記載し，医師，被験者各々が署名する．そして，1部コピーし，1部は被験者本人に手渡し，同意書の原本は試験実施機関で定められた保管場所に保管する．

同意説明における内容は以下の通りである．

1) 本研究が臨床試験であること

2) 本研究のデザインおよび根拠（意義，登録数，必要性，目的など）

3) プロトコ－ル治療の内容

4) プロトコ－ル治療により期待される効果

5) 予期される有害事象，後遺症とその対処法について

予期される有害事象の程度と発現割合，及びそれらが生じた際の対処法について．

6) 費用負担と補償

本研究で行われるマインドフルネス瞑想および頭部MRI撮像は、研究費により無償で行い、有害事象が起こる可能性は極めて低いと考えられる。本研究中のパーキンソン病診療は、保険償還で認められている医薬(or 医療機器)を用いて行われ，実施する検査も日常診療の範囲内であることから，通常通りの保険診療とする．従って，特別な補償は行われず，通常の診療を受けた際に発症した健康被害や医療事故と同じ扱いになる．同意取得時のパーキンソン病治療薬を研究責任（分担）者の指示に従って適正に使用し，重篤な有害事象，またはその他の副作用に対する治療は日常診療の場合と同様に，通常の保険診療で行う．

7) 本研究に参加することで被験者に予想される利益と可能性のある不利益

本研究に参加することによって享受できると思われる利益と被る可能性のある不利益．

8) 同意拒否と同意撤回

試験参加に先立っての同意拒否が自由であることや，いったん同意した後の同意の撤回も自由であり，それにより不当な診療上の不利益を受けないこと．

9) 有害事象が生じた場合の対処

研究の実施に起因して有害事象が発生し，被験者に健康被害が生じた場合には，研究責任者又は研究分担者は，適切な治療その他必要な措置を含めた最善の処置を行う．

10) プライバシー保護

氏名や個人情報は守秘されるための最大限の努力が払われること．

11) 質問の自由

研究事務局の連絡先を文書で知らせ，試験や治療内容について自由に質問できることを説明する．

12) 研究終了後の資料の保存

被験者本人が試験参加に同意した場合，付表の同意書を用いて被験者本人による署名を得る．担当医は同意書に説明を行った医師名と説明日，説明を受け同意した被験者名，同意日の記載があることを確認する．

## 11-3. 個人情報の保護

本試験に関わる全ての関係者は，被験者の個人情報を厳格に保護する．関係者は，被験者の個人情報およびプライバシー保護に最大限の努力を払い，本試験を行う上で知り得た個人情報を正当な理由なく漏らしてはならない．関係者がその職を退いた後も同様とする．

被験者の同意取得後はデータ管理，症例の取り扱いにおいては全て被験者識別コード又は登録番号により管理され，被験者識別コードおよび登録番号と氏名の対応表、および氏名が記載された同意書は脳神経内科の施錠可能な書類保管庫に厳重に保管する．また，公表に際しては個人情報が直接公表されることがない等，被験者の個人情報の保護については十分に配慮する．

## 11-4. 遺伝的特徴等に関する重要な知見

　本試験では被験者等に健康，子孫に受け継がれ得る遺伝的特徴等に関する重要な知見がない．

## 11-5. 被験者情報の開示及び被験者からの問い合わせへの対応

　被験者本人よりプライバシーに関する情報の開示などを求められた場合の対応者は，原則として当該被験者の研究機関の研究者等(研究責任医師，担当医師)とする．

　プライバシーポリシーに関する一般的な問い合わせや苦情は，下記にて，郵送，電子メール，Faxのいずれかの方法で受け付ける．

　郵送先：〒641-8510 和歌山県和歌山市紀三井寺811-1

　e-mail：jinsoo@wakayama-med.ac.jp

　電話番号：073-441-0655

　Fax番号：073-441-0655

# 12. 補償

　研究の実施に起因して有害事象が発生し，被験者に健康被害が生じた場合には，研究責任者又は研究分担者は，適切な治療その他必要な措置を含めた最善の処置を行う．

# 13. 被験者等に対する謝礼・経済的負担

　被験者に対する謝礼は支払わない．また，本研究は通常の保険診療の範囲内で行われるため，本研究に係る被験者に対する追加の費用負担はない．頭部MRI撮像に関しては研究費にて行う．

# 14. 研究の費用負担(資金源)

　本研究は，講座研究費およびTakeda Japan Medical Affairs Funded Research Grant 2018によって実施することとする．なお，頭部MRI以外の本研究に関する医療費はすべて通常の健康診療の範囲内で行われ，臨床試験中の観察，検査，治療，その他の使用薬剤等にかかる費用の被験者の自己負担分は，被験者が支払うこととする．

# 15. プロトコールの変更及び試験中止・終了

## 15-1 プロトコールの変更

　本研究中に実施計画書の変更の必要性が生じた場合は，研究責任者は変更内容を決定し，速やかに変更内容とその理由を研究分担者に文書により報告する．実施計画書の重大な変更が行われる場合には，研究責任者は，倫理審査委員会及び学長に報告し，変更の了承を得る．

## 15-2 プロトコールの終了

　データが固定された時点で研究の終了とする．研究責任者は，研究が終了したことを研究分担者に報告する．

## 15-3 プロトコールの中止

研究全体の中止規則

1) 研究責任者からの研究進捗報告を評価した結果，症例登録の遅れ，研究計画書逸脱の頻発などの理由により，研究の完遂が困難と判断された場合

2) 本研究の安全性に重大な問題があると判定された場合

3) 論文や学会発表など，本研究以外から得られた関連情報を評価した結果，本研究の安全性に問題があると判断された場合，又は研究継続の意義がなくなったと判断された場合

研究全体の中止決定の手順

研究責任者は，和歌山県立医科大学 倫理審査委員会による審査を依頼し，報告を行わなければならない．

研究責任者が研究全体の中止の決定を行った場合は，その理由および以後の対応を直ちに研究分担者に連絡する．連絡を受けた研究分担者は，被験者に研究全体の中止およびその理由を伝え，直ちに然るべき対応を行う．

# 16. 試験管理

## 16-1. モニタリング

　モニタリングは，患者の人権，安全性および福祉が保護されていること，本試験が最新の研究計画書・標準業務手順書等を遵守して実施されていることを確認する．原則として年1回定期モニタリングを行う．モニタリング担当者が作成する定期モニタリングは，モニタリング報告書にまとめ，研究事務局，研究責任者に提出され，記録される．

(1) モニタリングの方法

　モニタリングは施設訪問モニタリングを基本とし，モニタリング担当者が定期的に実施する．施設訪問モニタリングは，収集された症例報告書およびその他の報告事項に基づいて，研究が安全かつ本研究実施計画書に従って実施されていることを確認するとともに，電子カルテの直接閲覧を通じて，症例報告書が適切に記載されているか否かを点検する．直接閲覧の症例数は，√(必要症例数)を基本として，ランダムに選択する．モニタリング担当者は，モニタリングの結果を研究責任者に報告する．施設訪問モニタリングの結果から，当該試験の実施体制に改善の必要性が判断された場合は，研究代表者は，適切に改善する．

(2) モニタリング項目

① 集積達成状況

② 症例報告書の適切性の検討

➂ 選択基準・除外基準の適合

④ 重篤な有害事象

⑤ プロトコール逸脱

⑥ プロトコール治療の中止，終了の理由

⑦ 症例の背景因子

⑧ その他，試験の進捗や安全性に関する問題点

## 16-2. 監査

　行わない．

# 17. 試料・情報等の保存

## 17-1. 原資料等の定義

　試験における原資料とは以下のものをいう．

① 被験者の同意および情報提供に関する記録．

② 診療記録，臨床検査データおよび画像検査フィルム等，症例登録票データの元となった記録．

なお，電子カルテに格納されたデータも原資料とみなす．

## 17-2. 実施医療機関における記録の保存

研究終了後，研究より得られた試料・情報等は匿名化し，情報は外部記憶装置に記録し鍵をかけて保存，試料も鍵をかけて保存する．論文発表から試料は5年保存し，情報は10年保存する．その後，特定の個人を識別することができないような適切な方法で各所属の手順に従って廃棄する．

① 原資料．

② 同意文書，その他実施研究機関に従事する者が作成した本試験に関する文書，またはその写し．

③ 試験実施計画書，倫理審査委員会から入手した研究の審査に関する文書，本研究の実施に際して入手した文書

④ その他の本研究に関わる業務の記録．

## 17-3. 原資料の直接閲覧・提供

　院内システム監査、モニタリングにおいて第3者による原資料の直接閲覧を伴う症例報告書の照合が行われる可能性がある．また，試験終了後，規制当局の指示・指導などにより，個人情報を除いた本試験データを規制当局に提供することがある．

## 17-4. データの二次利用

　MRI研究は解析ソフトウェアの開発によって進歩を遂げている分野である。今回撮像された頭部MRI画像データを用いて、将来的に開発された新しい解析法によって新たな知見が得られる可能性がある。よって同意を得られた参加者に対して、MRI画像データおよび必要な臨床データについて、個人を特定できない連結不可能匿名化を行い、データの二次利用のために和歌山県立医科大学脳神経内科のMRIデータベースに登録し、保存する。

# 18. 研究に関する情報公開

　研究実施に先立ち，研究責任者が事前にUMIN臨床試験登録システム(UMIN-CTR)に登録する．また，研究終了後に同システムに研究終了結果を登録する．また，研究終了後，速やかに学会発表あるいは論文によって結果を公表する．

# 19. 研究組織

1. 研究責任者

| 氏　名 | 機関名，部署・所属，役職 |
| --- | --- |
| 髙　真守 | 和歌山県立医科大学　脳神経内科　助教 |

1. 統計解析担当者

| 氏　名 | 機関名，部署・所属，役職 |
| --- | --- |
| 吉川　隆範 | 和歌山県立医科大学附属病院　臨床研究センター・データーセンター部門　助教 |

1. データマネジメント責任者

| 氏　名 | 機関名，部署・所属，役職 |
| --- | --- |
| 髙　真守 | 和歌山県立医科大学　脳神経内科　助教 |

1. モニタリング担当者

| 氏　名 | 機関名，部署・所属，役職 |
| --- | --- |
| 髙橋　麻衣子  高　真守 | 和歌山県立医科大学　脳神経内科　学内助教  和歌山県立医科大学　脳神経内科　助教 |

1. 試料・情報等に関する管理責任者

| 氏　名 | 機関名，部署・所属，役職 |
| --- | --- |
| 髙　真守 | 和歌山県立医科大学　脳神経内科　助教 |

1. 個人情報分担管理者

| 氏　名 | 機関名，部署・所属，役職 |
| --- | --- |
| 髙　真守 | 和歌山県立医科大学　脳神経内科　助教 |

1. 研究事務局

| 名称 | 所在地，電話番号，FAX番号，E-mail |
| --- | --- |
| 和歌山県立医科大学　脳神経内科 | 和歌山県和歌山市紀三井寺811-1  TEL/FAX: 073-441-0655  E-mail: jinsoo@wakayama-med.ac.jp |

1. 患者相談窓口

| 名称 | 所在地，電話番号，FAX番号，E-mail |
| --- | --- |
| 和歌山県立医科大学　脳神経内科 | 和歌山県和歌山市紀三井寺811-1  TEL/FAX: 073-441-0655  E-mail: jinsoo@wakayama-med.ac.jp |

# 20. 参考文献

1. Bilevicius E, Smith SD, Kornelsen J. Resting-State Network Functional Connectivity Patterns Associated with the Mindful Attention Awareness Scale. Brain Connect. 2018;8(1):40-8.

2. Brewer JA, Worhunsky PD, Gray JR, Tang YY, Weber J, Kober H. Meditation experience is associated with differences in default mode network activity and connectivity. Proc Natl Acad Sci U S A. 2011;108(50):20254-9.

3. Carriere N, Lopes R, Defebvre L, Delmaire C, Dujardin K. Impaired corticostriatal connectivity in impulse control disorders in Parkinson disease. Neurology. 2015;84(21):2116-23.

4. Franco C, Amutio A, Lopez-Gonzalez L, Oriol X, Martinez-Taboada C. Effect of a Mindfulness Training Program on the Impulsivity and Aggression Levels of Adolescents with Behavioral Problems in the Classroom. Front Psychol. 2016;7:1385.

5. Gilmartin H, Goyal A, Hamati MC, Mann J, Saint S, Chopra V. Brief Mindfulness Practices for Healthcare Providers - A Systematic Literature Review. Am J Med. 2017;130(10):1219 e1- e17.

6. Khoury B, Lecomte T, Fortin G, Masse M, Therien P, Bouchard V, et al. Mindfulness-based therapy: a comprehensive meta-analysis. Clin Psychol Rev. 2013;33(6):763-71.

7. Kwok JYY, Kwan JCY, Auyeung M, Mok VCT, Lau CKY, Choi KC, et al. Effects of Mindfulness Yoga vs Stretching and Resistance Training Exercises on Anxiety and Depression for People With Parkinson Disease: A Randomized Clinical Trial. JAMA Neurol. 2019.

8. Marin-Lahoz J, Pagonabarraga J, Martinez-Horta S, Fernandez de Bobadilla R, Pascual-Sedano B, Perez-Perez J, et al. Parkinson's Disease: Impulsivity Does Not Cause Impulse Control Disorders but Boosts Their Severity. Front Psychiatry. 2018;9:465.

9. McLean G, Lawrence M, Simpson R, Mercer SW. Mindfulness-based stress reduction in Parkinson's disease: a systematic review. BMC Neurol. 2017;17(1):92.

10. Piet J, Hougaard E. The effect of mindfulness-based cognitive therapy for prevention of relapse in recurrent major depressive disorder: a systematic review and meta-analysis. Clin Psychol Rev. 2011;31(6):1032-40.

11. Sheline YI, Barch DM, Price JL, Rundle MM, Vaishnavi SN, Snyder AZ, et al. The default mode network and self-referential processes in depression. Proc Natl Acad Sci U S A. 2009;106(6):1942-7.

12. Sheline YI, Price JL, Yan Z, Mintun MA. Resting-state functional MRI in depression unmasks increased connectivity between networks via the dorsal nexus. Proc Natl Acad Sci U S A. 2010;107(24):11020-5.

13. Sperduti M, Martinelli P, Piolino P. A neurocognitive model of meditation based on activation likelihood estimation (ALE) meta-analysis. Conscious Cogn. 2012;21(1):269-76.

14. Strauss C, Cavanagh K, Oliver A, Pettman D. Mindfulness-based interventions for people diagnosed with a current episode of an anxiety or depressive disorder: a meta-analysis of randomised controlled trials. PLoS One. 2014;9(4):e96110.

15. Szewczyk-Krolikowski K, Menke RA, Rolinski M, Duff E, Salimi-Khorshidi G, Filippini N, et al. Functional connectivity in the basal ganglia network differentiates PD patients from controls. Neurology. 2014;83(3):208-14.

16. Weintraub D. Dopamine and impulse control disorders in Parkinson's disease. Ann Neurol. 2008;64 Suppl 2:S93-100.

17. Weintraub D, Koester J, Potenza MN, Siderowf AD, Stacy M, Voon V, et al. Impulse control disorders in Parkinson disease: a cross-sectional study of 3090 patients. Arch Neurol. 2010;67(5):589-95.

18. Wilson AD, Roos CR, Robinson CS, Stein ER, Manuel JA, Enkema MC, et al. Mindfulness-based interventions for addictive behaviors: Implementation issues on the road ahead. Psychol Addict Behav. 2017;31(8):888-96.

19. Xue J, Zhang Y, Huang Y. A meta-analytic investigation of the impact of mindfulness-based interventions on ADHD symptoms. Medicine (Baltimore). 2019;98(23):e15957.

20. Yang J, Liu Z, Liu S, Li L, Zheng L, Guo X. The emotional stability of elders with tai chi experience in the sequential risk-taking task. Psych J. 2019.

21. Zhao J, Tomasi D, Wiers CE, Shokri-Kojori E, Demiral SB, Zhang Y, et al. Correlation between Traits of Emotion-Based Impulsivity and Intrinsic Default-Mode Network Activity. Neural Plast. 2017;2017:9297621.
