# Supplementary material for: A single-arm open-label pilot study of brief mindfulness meditation to control impulsivity in Parkinson’s disease
Source: PLoS One. 2022 Apr 6;17(4):e0266354. doi: 10.1371/journal.pone.0266354 (PMC8985985; doi:10.1371/journal.pone.0266354)
Supplement: S3 File — (DOCX) [file pone.0266354.s005.docx]

|  |
| --- |
| Research protocol  A pilot study of mindfulness meditation on trait impulsivity of Parkinson's disease:  A single-arm, open-label, pilot study  (MIMP study) |
|  |
| **[Principal Investigator]**  Jinsoo Koh  　Department of Neurology, Wakayama Medical University  　811-1 Kimiidera, Wakayama City, Wakayama Prefecture  　TEL: 073 - 441 - 0655  　jinsoo@wakayama-med.ac.jp    **[Research Secretariat]**  　Jinsoo Koh  　Department of Neurology, Wakayama Medical University  　811-1 Kimiidera, Wakayama City, Wakayama Prefecture  　TEL: 073 - 441 - 0655  　jinsoo@wakayama-med.ac.jp |
| v1.1 |

Change History

| Version | date | Changes | Reasons |
| --- | --- | --- | --- |
| 1.0 | 06.18.2019 | Enactment | - |
| 1.1 | 10.23.2019 | 5-3. Precautions regarding registration (5) within 28 days from registration   → within 70 days | To allow participants to enroll in the next session while the current session is in progress. |
|  |  | 9-2. schedule  Observation period: within 28 days → within 70 days | As above |

**Abstract**

| item | Contents |
| --- | --- |
| Purpose | To investigate the effectiveness of mindfulness meditation for trait impulsivity in patients with Parkinson's disease (PD). |
| Research subjects | Patients with PD. |
| Inclusion criteria | 1. Clinically established or probable PD diagnosed by MDS clinical diagnostic criteria for Parkinson's disease.  2. 20 years or older.  3. Patients providing informed consent. |
| Exclusion criteria | 1. Severe dementia or hallucination/delusion.  2. Severe hearing loss or severe visual impairment.  3. Disturbance in consciousness.  4. Participating in other intervention trials.  5. Change in antiparkinsonian drugs within 28 days before enrollment.  6. New antipsychotics or antidepressants within 84 days before enrollment.  7. Symptomatic stroke or encephalitis within 84 days before enrollment.  8. Daily meditation or yoga. |
| Informed consent | Prior to the start of the study, the investigating doctor explains it to the participant in an easy-to-understand manner using the explanation document and consent form. The participant is given sufficient time to think and is confirmed to have understood the contents of the test well. The consent form requires the date of consent and signatures from the investigating doctor who gave the explanation and from the participant who gave consent after the explanation. A copy of the consent form is then given to the participant and the original is retained. |
| Intervention method | Mindfulness meditation.  Participants undergo a weekly group session for approximately 30 min of mindfulness meditation for 8 weeks, and are required to practice meditation daily for 15 min on 6 or 7 days per week. Mindfulness meditation includes body scans, mindfulness movements, and controlled breathing. The participants are given instruction papers in each group session for daily home-based practice, and complete an implementation record. In addition to regular meditation, participants also receive instructions on meditation techniques during daily activities, such as eating meditation and walking meditation. During the period from registration to the final evaluation, the antiparkinsonian, antipsychotic, or antidepressant drugs used by participants are not permitted to be changed. |
| Research method  (Treatment schedule, etc. ) | We recruit patients with PD visiting our institute, explain the purpose and methods of this trial, and request research participation. Participants are registered after providing written informed consent. After registration, they are evaluated by cognitive test (MMSE), motor symptom evaluation (UPDRS part 3, Hoehn- Yahr), MRI, questionnaire evaluations (BIS-11, QUIP-RS-J, HADS, PDQ-8, MAAS, NPI-Q). For cases with wear-off phenomenon, MMSE, UPDRS, and MRI should be performed in “ON” state. MRI can be skipped in difficult cases, such as for patients with claustrophobia.  Group sessions of mindfulness meditation are performed once a week under the guidance of a professional clinical psychologist, including approximately 30 minutes of guidance and 15 minutes of mindfulness meditation. Mindfulness meditation includes body scans and controlled breathing. Participants are required to practice meditation daily for 15 minutes on 6 or 7 days per week. The participants receive instruction papers in each group session for daily home-based practice and complete an implementation record. Clinical evaluations are performed in week 2 and week 8, and MRI is conducted within 14 days after week 8 evaluation. Daily mindfulness meditation continues until the final MRI. For evaluation items and schedule, refer to “#9, Inspection items and schedule." |
| Concomitant prohibited drugs | From the date of registration to the end of study, new additions and changes to antiparkinsonian drugs, antipsychotic drugs, and antidepressants are prohibited. |
| Outline of observation / inspection schedule | - Demographics: age, gender, height, weight, medical history (duration, age at onset, etc.), medication, past history and complications, and cognitive function (MMSE). - Motor symptoms: MDS-UPDRS, Hoehn-Yahr scale. - Questionnaires: Impulsivity Evaluation Scale (BIS-11), Impulse Control Disorder Scale (QUIP-RS-J), Depression and Apathy Evaluation Scale (HADS), QOL Evaluation Scale (PDQ-8), Mindfulness Evaluation Scale (MAAS) is performed after the observation period, at 2 weeks and at 8 weeks. If there are any caregivers, NPI- Q is also performed as an evaluation of psychiatric symptoms. - MRI is performed twice, before the intervention and then within 14 days after the end of the study. - Evaluation of achievement rate of mindfulness meditation using implementation record (15 minutes, 6 or 7 days per week) |
| Primary outcome | BIS-11 (Barratt Impulsiveness Scale) score reduction following 8 weeks of mindfulness meditation. |
| Target number of cases | 20 |
| Subject registration period | Approval date - 08/31/2021 |
| Research period | Approval date – 08/31/2024 |

**1. Research purpose and significance**

**1-1. Purpose**

We aim to investigate the effectiveness of mindfulness meditation for trait impulsivity in patients with Parkinson's disease (PD).

**1-2. Significance**

This clinical study provides data on whether mindfulness meditation can control the impulsivity of PD. In addition, it will be the preliminary data for phase III clinical trials.

**2. Background**

PD is the second most common neurodegenerative disease after Alzheimer's disease, affecting 100~150/100,000 people. PD is caused by degeneration of dopaminergic neurons and presents as motor symptoms, such as akinesia, tremors, and rigidity. No radical cure has yet been developed; the main treatment is dopamine replacement therapy, such as levodopa or dopamine agonists, which is also described in clinical practice guidelines. Conversely, it has been suggested that motor symptoms are only one aspect of PD, and non-motor symptoms have also been highlighted. Psychiatric symptoms, such as depression, apathy, anhedonia, and impulse control disorders (ICDs), are major problems that can reduce quality of life. ICDs have been reported to occur in about 13% of PD patients, and include pathological gambling, binge eating, excessive shopping and hypersexuality. The ICD related symptoms include punding and dopamine dysregulation syndrome. To improve ICDs, dopamine replacement therapy needs to be reduced. Meanwhile, dose reduction of dopamine agonist can induce dopamine agonist withdrawal syndrome (DAWS).

The pathophysiological mechanism of ICDs in PD has not been elucidated. In PD, the dopaminergic neurons in the substantia nigra pars compacta are reduced, but the dopaminergic neuron in the ventral tegmental area, which plays an important role in reward and goal-directed behavior, are relatively preserved. Dopamine replacement therapy has been reported to cause ICDs due to hyperexcitability from the ventral tegmental area to the ventral striatum and frontal lobe. Owing to individual differences in the likelihood of developing ICDs, some vulnerabilities in the reward system have been shown in patients with ICD. In addition, patients with high impulsivity have more severe ICDs. The trait impulsivity itself may therefore be a psychological symptom of PD.

In recent years, mindfulness meditation has attracted attention as a non-pharmacotherapy in the field of psychiatry. The mindfulness-based stress reduction (MBSR) developed by Jon Kabat-Zinn et al. is an eight-week secular course of practice derived from Buddhist meditations methods. Based on MBSR, a short version (15 minutes) and MBCT have been developed. These methods have been shown to improve depression and anxiety, and also prevent the recurrence of depression. There have been recent reports of improved symptoms of attention deficit hyperactivity disorder, drug abuse and decreased impulsivity in adolescents. On the other hand, in a phase III trial, the effect of mindfulness yoga on depression has been shown in patients with PD. In this context, mindfulness meditation is thus a promising candidate treatment for ICDs or controlling impulsivity. Its effectiveness has not been fully investigated, however, so we planned a single-arm intervention study as a pilot study to investigate whether the impulsivity of PD is improved by mindfulness meditation.

Furthermore, to investigate the pathophysiology of impulsivity and changes of brain function by mindfulness meditation, we also evaluate structural and functional MRI. Recent developments in resting-state functional MRI (rs-fMRI) analysis have made it possible to non-invasively measure functional connectivity of the brain. There have been various reports related to mindfulness, impulsivity, and rs-fMRI: the association between default mode network (DMN) and impulsivity, the association between mindfulness scale (MAAS) and attention network, or inter-large-scale networks; suppressing effect of DMN hyperexcitability by meditation. The functional connectivity between the striatum and the motor system is reduced in patients with PD, which can be improved by dopamine replacement therapy. Regarding ICDs, dysfunction in the ventral striatum, which controls the reward system, has been reported, although with some controversy. Some reports suggest hypoactivation, while others suggest hyperactivation. Furthermore, one report suggests a decreased functional connectivity between association striatum and inferior temporal cortex. In other words, there may be a wide range of dysfunctions beyond the reward system. In addition to dysfunction, MBSR has also been reported to increase gray matter volume in the amygdala and hippocampus in patients with PD. Based on the results of previous studies, as a secondary exploratory outcome we will conduct rs-fMRI and structural MRI before and after mindfulness meditation for PD.

**3. Research plan**

**3-1. Study population**

The target is patients with Parkinson's disease who visit our department.

**3-2. Design**

(1) Type of test

Single-center, single-arm, open-label, pilot study

(2) Intervention

Mindfulness meditation

(3) Explanation of test design

This pilot study aims to explore the effectiveness of mindfulness meditation for impulsivity of PD.

**3-3. Study period**

Study period: 2019/9/1- 2024/8/31 (5 years)

Registration period: 2019/9/1-2021/8/31

**3-4. Target registration number**

The target number is 20 patients with PD.

**3-5. Mindfulness meditation and measurement of outcomes**

3-5-1. Mindfulness meditation and clinical evaluation

The method of mindfulness meditation is as a 15-minute short version^5^, based on conventional mindfulness-based stress reduction (MBSR). Participants take part in a weekly group session (approximately 30 min) of mindfulness meditation for 8 weeks, and are required to practice meditation daily for 15 min on 6 or 7 days per week. Mindfulness meditation includes body scans and controlled breathing. The participants receive instruction papers in each group session for daily home-based practice, and fill in an implementation record. Mindfulness meditation is performed in the resting or lying position, so minimally invasive and safe. In addition to regular meditation, the participants also receive instructions on meditation techniques during daily activities, such as eating meditation and walking meditation. During the period between registration and the final evaluation, the participants are prohibited from changing antiparkinsonian, antipsychotic, or antidepressant drugs. Evaluation comprises the following questionnaire and a medical examination by an investigating doctor.

3-5-2. MRI

MRI will be performed at the Wakayama Minami Radiology Clinic. T1- and T2- weighted, resting-state functional MRI are taken. The imaging time is about 30 minutes. The procedure is the same as for clinical use and the cost is covered by research funding. MRI is performed twice: during the observation period and within 14 days of the final evaluation.

**3-7. Expected benefits**

By participating in this study, there may be improvement in impulse control disorder, impulsivity or depression. In addition, it may bring unexpected health benefits, such as improved concentration. It is non-invasive and is very unlikely to have side effects.

**3-8. Expected risks and disadvantages**

Participation in this study requires group lessons each week and 15 minutes of self-practice daily at home. Participants are responsible for the cost of travel to the hospital. Drug changes are restricted during the study period. The physical burden of mindfulness meditation is negligible. For participants who have difficulty sitting for 15 minutes, it is possible to perform the meditation in a lying position or other posture.

**4. Eligibility criteria**

**4-1 Inclusion Criteria**

1. Clinically established or probable PD diagnosed by MDS clinical diagnostic criteria for Parkinson's disease.

2. 20 years or older.

3. Patients providing informed consent.

**4-2 Exclusion criteria**

1. Severe dementia or hallucinations/delusion.

2. Severe hearing loss or severe visual impairment.

3. Disturbance in consciousness.

4. Participating in other intervention trials.

5. Changed antiparkinsonian drugs within 28 days before enrollment.

6. New antipsychotics or antidepressants within 84 days before enrollment.

7. Symptomatic stroke or encephalitis within 84 days before enrollment.

8. Daily meditation or yoga.

**5. Registration**

**5-1. Procedure**

The investigator confirms that the participant meets all eligibility criteria, fills in the necessary items on the registration form, and saves it in a file. The investigator will start treatment after registration is complete.

**5-2. Contact**

If participants have any opinions or questions regarding registration, they should contact the registration office:

Department of Neurology, Wakayama Medical University

#640 - 8510 Wakayama Kimiidera 811-1

Tel: 073-441-0655

Fax: 073-441-0655

E-mail: jinsoo@wakayama-med.ac.jp

**5-3. Precautions**

1. Registration after the start of protocol treatment is not allowed in any case.
2. The investigators should contact the registration office if they have any questions.
3. Except in cases where consent is withdrawn, subjects will not be unregistered.
4. If incorrect registration or duplicate registration is found, the registration office is contacted immediately.
5. Intervention is started within 70 days of enrollment.

**6. Procedure**

**6-1. Research flow**

We recruit patients with PD visiting our institute. We explain the purpose and methods of this trial, and request research participation. Participants are registered after written informed consent. After registration, participants are evaluated by cognitive test (MMSE), motor symptom evaluation (UPDRS part 3, Hoehn-Yahr), MRI, and questionnaire evaluations (BIS-11, QUIP-RS-J, HADS, PDQ-8, MAAS, NPI-Q). For cases with wear-off phenomenon, MMSE, UPDRS, and MRI should be performed in “ON” state. MRI can be skipped in difficult cases, such as for patients with claustrophobia.

Group mindfulness meditation sessions are performed once a week under the guidance of a professional clinical psychologist, including approximately 30 minutes of guidance and 15 minutes of mindfulness meditation. Mindfulness meditation includes body scans and controlled breathing. Participants are required to practice meditation daily for 15 minutes on 6 or 7 days per week. The participants are given instruction papers in each group session for daily home-based practice, and fill in an implementation record. Clinical evaluations are performed in week 2 and week 8, and MRI is conducted within 14 days after week 8 evaluation. Daily mindfulness meditation continues until the final MRI. For evaluation items and schedule, refer to “#9, Inspection items and schedule."

Concomitant prohibited drugs.

From the date of registration to the end of study, new additions and changes to antiparkinsonian drugs, antipsychotic drugs, and antidepressants are prohibited.

**6-2. protocol discontinuation, completion criteria**

　If any of the following items apply, the survey (evaluation / observation) of the subject will be discontinued.

1) Doctor's judgment

・ When it is judged that the underlying disease worsens and treatment should be discontinued.

・ When an adverse event occurs and it is judged that treatment should be stopped.

・ In addition, when it is judged that it is not possible to continue as the subject of this research.

2) When the subject withdraws consent.

3) Non-compliance with research protocol.

If it is found that the eligibility criteria are not met (if eligibility criteria were not met after registration, it will not be considered as discontinuation and will be regarded as "ineligible after registration" and the research participation will be terminated).

4) Other.

If the subject dies.

**7. Adverse events**

Adverse events are any unfavorable events that occur in the subject within 1 week after the end of the protocol treatment. A clinically significant exacerbation of symptoms that existed prior to this study is also an adverse event. Physiological changes that are considered to have no clinical significance in terms of frequency or degree are not considered as adverse events. If any adverse events are observed in the subject, the investigator should immediately ensure the safety of the subject and provide appropriate treatment, and describe the details in the case report form.

**7-1. Basic matters**

To ensure the safety of subjects, investigators must adhere to the following basics:

1) The investigators must comply with subject inclusion and exclusion criteria.

2) If the subjects are treated by a doctor other than the investigators of this study, they will be notified about participation in this study and its contents.

3) Continuing adverse events at the end of this study will be followed up as far as possible until they disappear or improve.

4) The subject is instructed to contact the investigator immediately if they feel their health is abnormal.

5) If an adverse event that requires treatment occurs, the researcher informs the participant and appropriate medical care is provided.

**7-2. Adverse events expected**

No particular adverse events are expected.

**7-3. Serious adverse events**

A serious adverse event is one of the following adverse events:

1. Death.
2. Life-threatening event.
3. Hospitalization or extension of hospital stay.
4. Permanent or significant disability/dysfunction.
5. Event(s) that cause birth defects in offspring.

**7-4. Adverse event reports**

If a serious adverse event occurs, the investigator must comply with the Wakayama Medical University Ethics Review Committee "Procedure for dealing with a serious adverse events in human medical research." The investigator should take appropriate measures regardless of the causal relationship with the study drug, and immediately report the contents in writing to the president of Wakayama Medical University. If an unexpected serious adverse event occurs, and if a direct causal relationship with the study cannot be ruled out, the president should promptly report to the Japanese Ministry of Health, Labor and Welfare.

**8. Outcome definition**

**8-1. Primary outcome**

The BIS-11 score reduction effect of eight weeks of mindfulness meditation.

**8-2. Secondary outcomes**

1. Questionnaires for clinical symptoms (week 0, week 2 and week 8)

1) Impulsivity scale: BIS-11.

2) Impulse control disorder scale: QUIP-RS-J.

3) QOL scale for Parkinson's disease: PDQ-8 .

4) Mindfulness scale: MAAS.

5) Depression and apathy: HADS.

6) Mental symptoms and care burden: NPI-Q.

2. Motor symptoms in Parkinson's disease (week 0, week 8)

1) MDS-UPDRS part 3.

2) Hohen-Yahr score.

3. Cognitive function (week 0)

1) Mini-Mental State Examination (MMSE).

4. MRI (week 0, week 8)

1) Resting-state functional MRI.

2) Voxel-based morphometry.

3) T1w / T2w ratio image.

**9. Inspection items and schedule**

**9-1. Inspection items and information to be reported**

- Demographics: age, gender, height, weight, medical history (duration, age at onset, etc.), medication, past history and complications, and cognitive function (MMSE).
- Motor symptoms: MDS-UPDRS, Hoehn-Yahr scale.
- Questionnaires: Impulsivity Evaluation Scale (BIS-11), Impulse Control Disorder Scale (QUIP-RS-J), Depression and Anxiety Evaluation Scale (HADS), QOL Evaluation Scale (PDQ-8), Mindfulness Evaluation Scale (MAAS) is performed after the observation period, 2 weeks and 8 weeks. If there are any caregivers, NPI- Q is also performed as an evaluation of psychiatric symptoms.
- MRI is performed twice: before the intervention and within 14 days after the end of the study.
- Evaluation of achievement rate of mindfulness meditation using implementation record (15 minutes, 6 or 7 days per week)

**9-2. Inspection / reporting schedule**

|  | Enrollment | Allocation | Post-allocation | | End of study |
| --- | --- | --- | --- | --- | --- |
| Timepoint | Within 70 days | 0 | Week 2 | Week 8 |  |
| Enrollment: |  |  |  |  |  |
| Eligibility evaluation | X |  |  |  |  |
| Informed consent | X |  |  |  |  |
| MMSE | X |  |  |  |  |
| Intervention: |  |  |  |  |  |
| Mindfulness Meditation |  |  |  |  |  |
| Evaluation: |  |  |  |  |  |
| BIS-11 | X |  | X | X |  |
| QUIP-RS-J | X |  | X | X |  |
| HADS | X |  | X | X |  |
| PDQ-8 | X |  | X | X |  |
| MAAS | X |  | X | X |  |
| Hoehen-Yahr | X |  |  | X |  |
| MDS-UPDRS part3 | X |  |  | X |  |
| NPI-Q | X |  | X | X |  |
| MRI | X |  |  |  | X |
| Diary evaluation |  |  | X | X |  |
| Adverse event |  |  |  |  |  |

**10. Statistics**

**10-1. Population**

The analysis target population in this study is defined as follows: if necessary, the principal investigator, the research secretariat, and the principal data management will discuss and make a decision before fixing the data.

Among the registered patients, the cases excluding duplicate and erroneous registrations are defined as "all registered cases". The group excluding "non-qualified cases" from all registered cases is defined as "all qualified cases".

- Effectiveness analysis target population.

All eligible patients who have undergone protocol treatment even once, excluding those who were found not to meet the eligibility criteria for this study after enrollment.

- Safety analysis target population.

Of all enrolled cases, those who received some or all of the protocol treatment.

**10-2. Rationale for sample size**

No reports have investigated the effects of mindfulness meditation on the impulsivity of PD. A report of mindfulness training on adolescents’ impulsivity reported that the BIS-11 score before the intervention was 82.49 ± 10.27, but decreased to 71.2 ± 9.16 after the intervention. In our previous study, patients with PD had a BIS-11 score of 62.6 ± 8.7, and healthy subjects had a BIS-11 score of 56.6 ± 10.6. We hypothesize that the change in BIS-11 is -10.0 ± 8.0 and the smallest clinically significant change is -5.0 by mindfulness meditation. If the significance level is 10% on both sides and the detection power is 80%, a sample size of 18 cases is required. Supposing there will be some ineligible cases, the required number of cases was set to 20 cases. The number of patients with PD in the Department of Neurology at Wakayama Medical University Hospital is about 300 per year, so it is estimated that the target number can be registered. SAS 9.4 was used to calculate the sample size.

**10-3. Primary outcome**

Calculate the two-sided 95% confidence interval for the BIS-11 change after 8 weeks and the *P*-value for the t-test. In addition, a summary statistic of the measured value and the amount of change at each time point is calculated.

**10-4. Secondary outcomes**

For each evaluation item, the actual measurement value is calculated at each time point as well as the summary statistic of the amount of change.

**11. Ethics**

**11-1. Rules**

All researchers carry out this study in accordance with the Declaration of Helsinki and the Ethical Guidelines for Medical Research in Humans.

**11-2. Informed Consent**

Prior to the start of the study, the investigating doctor explains it to the participant in an easy-to-understand manner using the explanation document and consent form. The participant is given sufficient time to think and they confirm they fully understand the contents of the test. The consent form requires date of consent, the signatures of the investigating doctor who gave the explanation and the agreeing participant after receiving the explanation. A copy of the consent form is given to the participants and the original is retained.

The contents of the consent explanation are as follows:

1) This study is a clinical trial.

2) Design and basis of this study (significance, number of registrations, necessity, purpose, etc.).

3) Contents of protocol treatment.

4) Expected effects of protocol treatment.

5) Expected adverse events, sequelae; and their treatments.

About the degree and incidence of expected adverse events, and what to do when they occur.

6) Cost and compensation.

The mindfulness meditation and MRI are funded by this study funds. The likelihood of adverse events is thought to be extremely low. The medical treatment and examinations under this study are covered by insurance, so they will be treated as usual and no special compensation in required if a medical accident occurs, no special compensation is required and it will be treated in typical way. Appropriate use of PD treatment at the time of obtaining consent according to the instructions of the researcher, and treatment for adverse events is performed as ordinary insurance medical treatment.

7) Expected benefits and potential disadvantages.

8) Refusal and withdrawal of consent.

Participants are free to refuse consent prior to participating in the study, and if they have consented, they can freely withdraw their consent, and thus do not suffer any unreasonable medical disadvantages.

9) What to do if an adverse event occurs.

If an adverse event occurs as a result of conducting the study, the investigator will take the best possible treatment.

10) Privacy protection.

Best effort will be made to keep the names and personal information of participants confidential.

11) Ask questions freely.

Provide written contact information for the research office and explain that you can freely ask questions about the study and treatment.

12) Preservation of materials after research.

**11-3. Protection of personal information**

All parties involved in this study will strictly protect the personal information of the subjects, will protect the subject's personal information and privacy as much as possible, and must not divulge any personal information that they have learned in conducting this test without a justifiable reason, even after retired from the position.

After obtaining consent from the subject, all data management and case handling are managed by the subject identification code or registration number. The subject identification code, the correspondence table between the registration number and the name, and the consent form with the name are strictly stored in the lockable document storage of the Department of Neurology. At the time of publication, due consideration will be given to protection of the subject's personal information, for example, personal information will not be published directly.

**11-4. Important findings regarding genetic characteristics, etc.**

In this study, there is no important knowledge about the health of the subjects and the genetic characteristics that can be inherited by offspring.

**11-5. Disclosure of subject information and response to inquiries from subjects**

Researchers respond when the subject requests disclosure of privacy-related information.　General inquiries and complaints regarding our privacy policy will be accepted by mail, email or fax listed below.

Address: 811-1 Kimiidera, Wakayama City, Wakayama Prefecture 641-8510

e-mail: jinsoo@wakayama-med.ac.jp

Phone number: 073-441-0655

Fax number: 073-441-0655

**12. Compensation**

If an adverse event occurs as a result of conducting the study and the subject suffers a health hazard, the principal investigator or coordinator of the research will take the best possible course, including appropriate treatment and other necessary measures.

**13. Reward and financial burden on subjects, etc.**

No reward will be paid to the subject. Also, since this study is conducted within the scope of normal insurance medical care, there is no additional cost burden for the subjects involved in this study. MRI is funded by research.

**14. Research cost burden (funding source)**

This research will be carried out with the Grants-in-Aid for Scientific Research and Takeda Japan Medical Affairs Funded Research Grant 2018. All medical expenses related to this study other than MRI are within the scope of normal health care. Subjects are responsible for paying their own expenses for medical examinations, treatments, and other drugs used during clinical trials.

**15. Protocol change and test cancellation / termination**

**15-1. Change of protocol**

If it becomes necessary to change the implementation plan during this research, the principal investigator decides the content of the change and promptly reports the content of the change and reason to the research coordinator in writing. If any significant change is made to the implementation plan, the principal investigator shall report to the Ethics Review Board and the president and obtain approval for the change.

**15-2. End of protocol**

The study ends when the data is fixed. The principal investigator reports to the research coordinator that the research has been completed.

**15-3. Discontinuation of protocol**

Rules for discontinuing the entire study

1) If it is judged in the research progress report from the principal investigator that it is difficult to complete the research due to delays in case registration, frequent deviations, etc.

2) If it is determined that there is a serious problem regarding the safety of this study

3) If it is judged that there is a problem with the safety of this research as a result of evaluating related information obtained from other research, such as papers and conference presentations, or if it is judged that the significance of continuing the research is lost.

Procedure for deciding to discontinue the entire study

The principal investigator must request a review by the Wakayama Medical University Ethics Review Committee and make a report. If the principal investigator decides to discontinue the entire research, the research coordinator will be notified immediately of the reason and subsequent actions. The research coordinator who is contacted informs the subject of the discontinuation of the entire study and the reason, and takes appropriate action immediately.

**16. Trial management**

**16-1. Monitoring**

Monitoring confirms that the human rights, safety and well-being of the patient are protected, and that this test is conducted in compliance with the latest research plan and standard work procedure manual. As a general rule, regular monitoring is performed once a year. Periodic monitoring prepared by the person in charge of monitoring is compiled into a monitoring report, submitted to the research secretariat and the principal investigator, and recorded.

(1) Monitoring method

Facility visit monitoring is regularly performed by the monitor. Based on the collected case reports and other reports, the monitor confirms that the study is safe and conducted in accordance with this study protocol, and the monitor also directly browses the electronic medical record to check whether the case report is properly described. The number of cases for direct browsing is randomly selected based on √ (number of required cases). The monitor reports the monitoring results to the principal investigator. If the results of facility visit monitoring determine the need for improvement in the implementation system of the study, the principal investigator will make appropriate improvements.

(2) Monitoring items

1. Achievement status.
2. The appropriateness of the case report form.
3. Conformity of inclusion / exclusion criteria.
4. Serious adverse events.
5. Protocol deviation.
6. Reasons for discontinuation and termination of protocol treatment.
7. Background factors of the case.
8. Other problems related to test progress and safety.

**16-2. Audit**

No audit is performed.

**17. Storage of samples, information, etc.**

**17-1. Definition of source material**

The source materials for the test are as follows:

(1) Records of subject consent and information provision.

(2) Records that are the basis of case registration form data, such as medical records, clinical examination data, and imaging examination films.

The data stored in the electronic medical record is also regarded as the source material.

**17-2. Preservation of records**

After the research is completed, the samples and information obtained from the research will be anonymized, the information will be recorded in an external storage device and stored with a lock, and the samples will also be stored with a lock. Samples are stored for 5 years and information is stored for 10 years after the publication of the paper. After that, it is disposed of according to the procedure of each affiliation in an appropriate way so that a specific individual cannot be identified.

1. Source material.
2. Consent form, documents related to this study prepared by those engaged in the research institute.
3. Test implementation plan, documents related to research review obtained from the Ethics Review Committee, documents obtained when conducting this research
4. Records of other work related to this research.

**17-3. Direct viewing and provision of original materials**

In-hospital system audits and monitoring may involve collation of case report forms with direct viewing of the source material by a third party. In addition, after the test is completed, the test data excluding personal information may be provided to the regulatory authority according to the instructions and guidance of the regulatory authority.

**17-4. Secondary use of data**

MRI research is an area of ​​progress made through the development of analysis software. Using the MRI data applied this study, there is a possibility that new findings can be obtained by a new analysis method developed in the future. Therefore, for participants who have given their consent, MRI and clinical data will be anonymized without connection, and then registered in the MRI database of the Department of Neurology, Wakayama Medical University for secondary use.

**18. Disclosure of information on research**

Prior to conducting the research, the principal investigator will register with UMIN-CTR. In addition, after the research is completed, the research completion results will be registered in the system. The research results will be presented at an academic conference, and be submitted to a scientific journal.

**19. Research organization**

1) Principal investigator

| Full name | Institution name, department / affiliation, job title |
| --- | --- |
| Jinsoo Koh | Assistant Professor, Department of Neurology, Wakayama Medical University |

2) Person in charge of statistical analysis

| Full name | Institution name, department / affiliation, job title |
| --- | --- |
| Takanori Yoshikawa | Assistant Professor, Clinical Research Center, Data Center Division, Wakayama Medical University Hospital |

3) Data management manager

| Full name | Institution name, department / affiliation, job title |
| --- | --- |
| Jinsoo Koh | Assistant Professor, Department of Neurology, Wakayama Medical University |

4) Monitor staff

| Full name | Institution name, department / affiliation, job title |
| --- | --- |
| Maiko Takahashi  Jinsoo Koh | Assistant Professor, Department of Neurology, Wakayama Medical University  Assistant Professor, Department of Neurology, Wakayama Medical University |

5) Manager of samples, information, etc.

| Full name | Institution name, department / affiliation, job title |
| --- | --- |
| Jinsoo Koh | Assistant Professor, Department of Neurology, Wakayama Medical University |

6) Personal information sharing manager

| Full name | Institution name, department / affiliation, job title |
| --- | --- |
| Jinsoo Koh | Assistant Professor, Department of Neurology, Wakayama Medical University |

7) Research Secretariat

| Name | Location, phone number, fax number, E-mail |
| --- | --- |
| Department of Neurology, Wakayama Medical University | 811-1 Kimiidera, Wakayama City, Wakayama Prefecture  TEL / FAX: 073-441-0655  E-mail: jinsoo@wakayama-med.ac.jp |

8) Patient consultation desk

| Name | Location, phone number, fax number, E-mail |
| --- | --- |
| Department of Neurology, Wakayama Medical University | 811-1 Kimiidera, Wakayama City, Wakayama Prefecture  TEL / FAX: 073-441-0655  E-mail: jinsoo@wakayama-med.ac.jp |

**20. References**

1. Bilevicius E, Smith SD, Kornelsen J. Resting-State Network Functional Connectivity Patterns Associated with the Mindful Attention Awareness Scale. Brain Connect. 2018; 8 (1): 40-8.

2. Brewer JA, Worhunsky PD, Gray JR, Tang YY, Weber J, Kober H. Meditation experience is associated with differences in default mode network activity and connectivity. Proc Natl Acad Sci US A. 2011; 108 (50): 20254- 9.

3. Carriere N, Lopes R, Defebvre L, Delmaire C, Dujardin K. Impaired corticostriatal connectivity in impulse control disorders in Parkinson disease. Neurology. 2015; 84 (21): 2116-23.

4. Franco C, Amutio A, Lopez-Gonzalez L, Oriol X, Martinez-Taboada C. Effect of a Mindfulness Training Program on the Impulsivity and Aggression Levels of Adolescents with Behavioral Problems in the Classroom. Front Psychol. 2016; 7: 1385 ..

5. Gilmartin H, Goyal A, Hamati MC, Mann J, Saint S, Chopra V. Brief Mindfulness Practices for Healthcare Providers -A Systematic Literature Review. Am J Med. 2017; 130 (10): 1219 e1-e17.

6. Khoury B, Lecomte T, Fortin G, Masse M, Therien P, Bouchard V, et al. Mindfulness-based therapy: a comprehensive meta-analysis. Clin Psychol Rev. 2013; 33 (6): 763-71.

7. Kwok JYY, Kwan JCY, Auyeung M, Mok VCT, Lau CKY, Choi KC, et al. Effects of Mindfulness Yoga vs Stretching and Resistance Training Exercises on Anxiety and Depression for People With Parkinson Disease: A Randomized Clinical Trial. JAMA Neurol. 2019.

8. Marin- Lahoz J, Pagonabarraga J, Martinez-Horta S, Fernandez de Bobadilla R, Pascual- Sedano B, Perez-Perez J, et al. Parkinson's Disease: Impulsivity Does Not Cause Impulse Control Disorders but Boosts Their Severity. Front Psychiatry. 2018; 9: 465.

9. McLean G, Lawrence M, Simpson R, Mercer SW. Mindfulness-based stress reduction in Parkinson's disease: a systematic review. BMC Neurol. 2017; 17 (1): 92.

10. Piet J, Hougaard E. The effect of mindfulness-based cognitive therapy for prevention of relapse in recurrent major depressive disorder: a systematic review and meta-analysis. Clin Psychol Rev. 2011; 31 (6): 1032-40.

11. Sheline YI, Barch DM, Price JL, Rundle MM, Vaishnavi SN, Snyder AZ, et al. The default mode network and self-referential processes in depression. Proc Natl Acad Sci U S A. 2009; 106 (6): 1942 -7.

12. Sheline YI, Price JL, Yan Z, Mintun MA. Resting-state functional MRI in depression unmasks increased connectivity between networks via the dorsal nexus. Proc Natl Acad Sci US A. 2010; 107 (24): 11020-5.

13. Sperduti M, Martinelli P, Piolino P. A neurocognitive model of meditation based on activation likelihood estimation (ALE) meta-analysis. Conscious Cogn. 2012; 21 (1): 269-76.

14. Strauss C, Cavanagh K, Oliver A, Pettman D. Mindfulness-based interventions for people diagnosed with a current episode of an anxiety or depressive disorder: a meta-analysis of randomized controlled trials. PLoS One. 2014; 9 (4 ) : e 96110.

15. Szewczyk-Krolikowski K, Menke RA, Rolinski M, Duff E, Salimi-Khorshidi G, Filippini N, et al. Functional connectivity in the basal ganglia network differentiates PD patients from controls. Neurology. 2014; 83 (3): 208 -14.

16. Weintraub D. Dopamine and impulse control disorders in Parkinson's disease. Ann Neurol. 2008; 64 Suppl 2: S 93-100.

17. Weintraub D, Koester J, Potenza MN, Siderowf AD, Stacy M, Voon V, et al. Impulse control disorders in Parkinson disease: a cross-sectional study of 3090 patients. Arch Neurol. 2010; 67 (5): 589 -95.

18. Wilson AD, Roos CR, Robinson CS, Stein ER, Manuel JA, Enkema MC, et al. Mindfulness-based interventions for addictive behaviors: Implementation issues on the road ahead. Psychol Addict Behav. 2017; 31 (8): 888 -96.

19. Xue J, Zhang Y, Huang Y. A meta-analytic investigation of the impact of mindfulness-based interventions on ADHD symptoms. Medicine (Baltimore). 2019; 98 (23 ): e 15957.

20. Yang J, Liu Z, Liu S, Li L, Zheng L, Guo X. The emotional stability of elders with tai chi experience in the sequential risk-taking task. Psych J. 2019.

21. Zhao J, Tomasi D, Wiers CE, Shokri- Kojori E, Demiral SB, Zhang Y, et al. Correlation between Traits of Emotion-Based Impulsivity and Intrinsic Default-Mode Network Activity. Neural Plast. 2017; 2017: 9297621 .

1
